# Differences in weight loss practices and perceptions between medalists and non-medalists in national-level adolescent Sanda athletes

**DOI:** 10.1371/journal.pone.0359088

**Published:** 2026-09-24

**Authors:** Xu Han, Jianpeng Hou, Carl Langan-Evans, Anthony Weldon, Jad Adrian Washif, Nemanja Lakicevic, Yukun Wu, Shaoyun Liu, Mengfan Li, Haidong Jiang, Yuming Zhong

**Affiliations:** 1 Sports Teaching and Research Department, Heilongjiang University of Chinese Medicine, Harbin, China; 2 School of Athletic Performance, Shanghai University of Sport, Shanghai, China; 3 School of Coaching, Shanghai University of Sport, Shanghai, China; 4 School of Sport and Exercise Sciences, Tom Reilly Building, Liverpool John Moores University, Liverpool, United Kingdom; 5 Centre for Life and Sport Sciences, Birmingham City University, Birmingham, United Kingdom; 6 Aston Villa Foundation, Birmingham, United Kingdom; 7 Sports Performance Division, Institut Sukan Negara (National Sports Institute of Malaysia), Kuala Lumpur, Malaysia; 8 Faculty of Psychology, Lomonosov Moscow State University, Moscow, Russia; 9 Federal Scientific Center of Psychological and Interdisciplinary Research, Moscow, Russia; 10 Dali University, Dali, China; 11 Shaolin Tagou Wushu School, Zhengzhou, China; 12 Wushu college, Henan University, Kaifeng, China; 13 School of Martial Arts, Shanghai University of Sport, Shanghai, China; University of Urbino: Universita degli Studi di Urbino Carlo Bo, ITALY

## Abstract

**Objectives:**

This study aimed to compare weight loss (WL) practices and perceptions between medalists and non-medalists among Chinese national-level adolescent Sanda athletes.

**Methods:**

A cross-sectional survey was conducted among national-level adolescent Sanda athletes using the Rapid Weight Loss Questionnaire (RWLQ). One hundred and sixty-four participants completed the questionnaire, with 158 valid responses obtained. Athletes were categorized as medalists or non-medalists based on their competitive achievements.

**Results:**

Overall, 79% of athletes (n = 125) reported intentional WL. In unadjusted analyses, three nominal associations were observed, with medalists reporting greater habitual WL%, greater habitual WL% during the 9–60 days prior to weigh-in, and lower habitual WR/WL before competition than non-medalists. However, none remained statistically significant after Benjamini–Hochberg false discovery rate (FDR) adjustment. No statistically significant difference were observed for the other WL practices and perceptions variables (all *p* > 0.05).

**Conclusions:**

WL practices and perceptions were similar between national-level medalists and non-medalists. Although three nominal associations were identified in the unadjusted analyses, none remained statistically significant after Benjamini–Hochberg FDR adjustment. The present findings therefore do not provide robust evidence of systematic differences in WL characteristics according to medal achievement. The high prevalence and substantial magnitude of WL observed in this population warrant attention, particularly among adolescent athletes.

## Background

Sanda is an unarmed combat sport (CS) derived from traditional wushu techniques, comprising punching, kicking, throwing, wrestling and defensive actions [1]. In recent years, Sanda has gained increasing global popularity, with the International Wushu Federation comprising 160 national and territorial members across five continents [2]. Major international competitions include the World Cup and World Championships, with Sanda included in the 2008 Olympic and 2014 Youth Olympic Games as an exhibition event, and scheduled to become an official event at the 2026 Youth Olympic Games [3]. To ensure fair competition, Sanda athletes compete within weight classes, with failure to meet the required body mass (BM) resulting in disqualification or reassignment to a higher weight category, as observed across weight-classified CS [4].

In CS, weight loss (WL) is highly prevalent [4,5] and represents not only a physiological process but also a performance-oriented behavioral practice aimed at meeting weight-class requirements and potentially optimizing competitive performance [6,7]. A substantial proportion of Sanda athletes engage in WL practices, with previous studies reporting that 56% of Chinese national- and international-level Sanda athletes and up to 90% of Brazilian national-level athletes engaged in WL [8,9]. Similarly, high WL prevalence (66–100%) has been consistently observed across other CS such as judo, mixed martial arts, and sambo [4,10]. Given both its high prevalence and potential for severe consequences (e.g., mortality) [11–13], WL in CS has received an increased research attention in recent decades [6–9,14–41]. These studies have provided important insights into WL behaviors, demonstrating, for example, greater WL magnitude in higher-level athletes and substantial variation across countries within the same sport [4].

For national-level athletes, competitive success is the primary objective, and many may be willing to accept potential health risks in pursuit of performance advantages [42]. Athletes commonly perceive WL as a necessary component of competition preparation, believing that it enhances performance and increases the likelihood of winning [6,7,34,42]. Despite this widespread belief, current evidence remains inconclusive regarding the relationship between WL practices and competitive outcomes, with some studies reporting associations [43–45] while others finding no clear relationship [46–51]. From a methodological perspective, research on WL in CS has generally followed two distinct approaches. One approach examines WL within a single competition cycle, typically focusing on pre-weigh-in WL and post-weigh-in weight regain (WR), with the aim of exploring the relationship between WL and immediate competitive results (win or lose). The other approach focuses on habitual WL practices, capturing athletes’ repeated WL behaviors across competitions and over time, thereby reflecting more stable behavioral patterns.

To date, most of the existing evidence on the relationship between WL practices and competitive outcomes is derived from competition-based studies adopting the former approach, often using post-weigh-in WR as a proxy for WL. While these studies provide valuable insights into the short-term effects of WL on performance, they are limited to isolated events and may not fully represent athletes’ long-term WL practices. In contrast, habitual WL behaviors reflect more stable and repeated decision-making processes influenced by accumulated experience, coaching influence, and competition demands. From a behavioral regulation perspective, these practices can be understood as ways of regulating BM to meet performance goals. Examining these long-term patterns may therefore provide a different perspective on the relationship between WL and competitive success, particularly when competitive achievement is operationalized as cumulative achievement (e.g., whether an athlete has attained a medal at the national level across their career) rather than the outcomes of a single competition event (e.g., the overall result of one tournament cycle from preliminary rounds to finals). However, existing research on long-term WL practices in CS has primarily been descriptive, focusing on comparisons across sex, age, weight class, or competitive level, and has not examined whether athletes within the same competitive level, and has provided limited evidence on whether athletes competing at the same level but differing in competitive achievement report different WL characteristics, practices, or perceptions [6–9,14–40].

This gap is particularly relevant in national-level adolescent athletes, a population characterized by both highly competitive demands and developmental vulnerability. At the national level, performance differences are often relatively small, meaning that marginal performance advantages may be decisive for competitive success. At the same time, adolescent athletes are undergoing growth and maturation and may therefore be more susceptible to the negative physiological and developmental consequences of repeated WL practices [52–55]. As a result, this population may be especially likely to engage in intensive WL behaviors while simultaneously being at greater risk of potential harm. Within this context, comparing athletes with and without a history of national-level medal achievement may help characterize whether WL practices and perceptions vary according to competitive achievement. Against this background, it remains unclear whether WL behaviors meaningfully differentiate competitive success within this population. Therefore, the aim of this study was to compare WL practices and perceptions between medalists and non-medalists among Chinese national-level adolescent Sanda athletes.

## Materials and methods

### Experimental approach to the problem

The study employed an observational cross-sectional approach using a questionnaire to ascertain the WL practices of Chinese national-level adolescent Sanda athletes. To target the relevant population, this study adopted purposive sampling and convenience sampling. The study adhered to the cross-sectional reporting guidelines of Strengthening of Reporting of Observational Studies in Epidemiology (STROBE) cross-sectional reporting guidelines (S1 File) [56].

### Participants

The recruitment of participants included online and offline recruitment. A convenience sample was recruited. All athletes who met the inclusion criteria and were accessible through the participating sports teams during the data collection period were invited to participate. No random sampling procedure was employed. For online recruitment, a questionnaire link was sent to the managers and coaches of Sanda sports teams through social media (WeChat, Tencent, China), requesting that the questionnaire be shared with their athletes [57]. Paper questionnaires were distributed for offline recruitment at the China’s largest Wushu training base, which represents the highest competitive level of Sanda in China. The Sanda sports team managers assisted in recruiting participants during daily sports team meetings. All athletes who agreed to participate in the offline questionnaire were gathered in the conference room to complete the questionnaire. If any questions were unclear, researchers were available to clarify the meaning of the items without directing or influencing participants’ responses. Prior to questionnaire administration, all participants were orally informed about the purpose and procedures of the study and questionnaire. Each participant signed the written informed consent, and verbal informed consent was obtained from their parents prior to the questionnaire administration for all athletes. No a priori sample size calculation was performed because this was an exploratory cross-sectional study. Instead, recruitment continued throughout the predefined data collection period to maximize participation from the target population. In total, 164 participants completed the questionnaire. The inclusion criteria for participants were: (i) provided consent to participate in the study, (ii) age 12–17 years [25], (iii) participation in at least one official Sanda competition in the last 12 months, (iv) participation in Sanda competition at national, (v) currently training as an athlete on a Sanda sports team, and (vi) does not compete in the 100 + kg division. The lead author (Xu Han) pre-checked all responses, and 6 responses were considered invalid, of which 2 participants were excluded because they did not meet criterion (ii) and 4 participants because they did not meet criterion (v). Approval for conducting the study was secured from the Ethics Committee of the ***Shanghai University of Sport (number of approval: 102772023RT170).

### Questionnaire development and administration

The WJX web platform (WENJUANXING, www.wjx.cn) was used to construct, distribute, and collect all online questionnaire responses. Responses from paper questionnaires were manually input into the WJX web platform by the lead author (Xu Han). Weight-loss practices and perceptions were assessed using an adapted version of the Rapid Weight Loss Questionnaire (RWLQ), originally developed and validated by Artioli et al. [58] and subsequently modified by Zhong et al. [6] for use among Chinese CS athletes. The modifications consisted of the addition of several descriptive items while preserving the original questionnaire structure. The questionnaire consisted of 30 questions and featured four sections: (i) general information, (ii) competition experience, (iii) WL history, and (iv) WL perception. Depending on the item, responses were recorded as numeric values, dichotomous (yes/no) responses, or categorical multiple-choice options. The section on WL history and perception was only available for those who had engaged in WL. Data collection occurred from 15 November 2024 to January 1, 2025.

### Statistical analyses

All analyses were performed using SPSS 27.0 (IBM Corp., Armonk, NY). Descriptive statistics were used to summarize all results. The Shapiro-Wilk test was applied to assess normality. With the exception of habitual WL%, all continuous variables tested deviated from a normal distribution. Normally distributed continuous variables are presented as mean ± standard deviation (SD), whereas non-normally distributed continuous variables are presented as median (interquartile range, IQR). Categorical variables are presented as frequencies (%). Between-group differences were analyzed using an independent-samples t-tests for habitual WL%, the Mann-Whitney U test for all other continuous variables, and Pearson’s chi-square test for categorical variables. When its assumptions were violated because of sparse expected cell frequencies, Fisher–Freeman–Halton exact tests were applied instead. Because multiple outcomes were examined, the potential for inflation of type I error was addressed using the Benjamini–Hochberg false discovery rate (FDR) adjustment as a sensitivity analysis. Both unadjusted and FDR-adjusted p-values were considered when interpreting the findings. In addition to p values, effect sizes were reported for all significant continuous variables. Cohen’s d was reported for variable analyzed using independent-samples t tests, whereas rank-biserial correlation (r_rb_) was reported for variables analyzed using the Mann–Whitney U test. Significance was accepted at *p* < 0.05.

Athletes were classified as medalists if they had won at least one medal in a national-level competition at any point in their competitive career prior to data collection. For athletes with multiple medals, classification was based on the presence (vs. absence) of any national-level medal, and medal count was not considered in group allocation.

The calculation formula for habitual WL% was habitual WL (kg)/off-season BM; highest WL% was highest WL (kg)/off-season BM; habitual WR before competition (%) was habitual WR before competition (kg)/(off-season BM-habitual WL [kg]); habitual WR/WL was habitual WR(kg) after weigh-ins/habitual WL(kg)

## Results

One hundred and fifty-eight national-level Sanda athletes (122 males, 36 females) participated in this study. Participants’ general information is presented in Table 1. There was only one significant difference in the number of competitions participated in last competitive season (*p* < 0.001) between those who engaged in WL and those who never engaged in WL (Table 1). This difference remained statistically significant after Benjamini–Hochberg FDR adjustment.

**Table 1:** General information of Chinese adolescent national level Sanda athletes (n = 158).

Variables	Presentation	All participants (n=158)	Participants who never engaged in (n=33)	Participants who engaged in (n=125)	P	Adjusted P
Age (years)	median (IQR)	15.5 (15-16)	16.0 (15-17)	15.0 (15-16)	0.809	0.913
Stature (cm)	mean±SD	170.2±7.7	169.0±7.1	170.8±7.9	0.913	0.913
Current body mass (kg)	mean±SD	59.9±10.1	59.0±9.1	60.2±10.6	0.696	0.913
Off-season body mass (kg)	mean±SD	59.3±10.2	59.1±9.4	59.4±10.6	0.561	0.898
Age began training in current sport (years)	median (IQR)	11 (10-13)	11 (10-12)	11 (10-13)	0.275	0.733
Age began competing in current sport (years)	median (IQR)	15 (14-16)	15 (14-16)	15 (14-15)	0.254	0.733
Competitions participated last competitive season (n)	median (IQR)	2 (1-2)	1 (1-2)	2 (1-2)	<0.001*	<0.008*
Medals gained during last competitive season (n)	median (IQR)	1 (0-1)	1 (1-1)	0 (0-1)	0.393	0.786

Note: * = differences between groups (P < 0.05). IQR, interquartile range. SD, standard deviation.

In total, 79% of participants (n = 125, 95 males, 30 females) reported intentionally performing WL for competition, and 63% had not changed their weight category in the past two years. The results of the participants’ WL history are shown in Table 2.

**Table 2:** Weight loss history (n = 125) of Chinese adolescent national level Sanda athletes who reported engaging in weight loss practices.

Variables	Presentation	Total (n = 125)	Medalist (n = 46)	Non-Medalist (n = 79)	P	Adjusted P
Age began WL (yr)	median (IQR)	15 (14-16)	15 (14-16)	15 (14-15)	0.209	0.418
Highest WL (kg)	mean±SD	6.3 ± 2.1	6.8 ± 1.7	6.0 ± 2.2	—	—
Highest WL (%)	median (IQR)	11.3 (8.6-13.3)	12.0 (9.9-13.3)	10.5 (8.2-13.3)	0.063	0.158
Habitual WL (kg)	mean±SD	5.1 ± 2.3	5.6 ± 1.9	4.8 ± 2.4	—	—
Habitual WL (%)	mean±SD	8.7 ± 3.9	9.6 ± 3.5	8.1 ± 4.0	0.043*	0.143
Habitual WL during 9–60 days prior to weigh-in (%)	median (IQR)	4.5 (2.2-7.7)	6.0 (3.1-8.4)	3.9 (2.0-6.5)	0.019*	0.095
Habitual WL during 2–8 days prior to weigh-in (%)	median (IQR)	2.4 (1.1-3.8)	2.3 (1.5-3.9)	2.4 (1.1-3.6)	0.563	0.780
Habitual WL during 1 days prior to weigh-in (%)	median (IQR)	0.5 (0.0-1.3)	0.6 (0.0-1.3)	0.5 (0.0-1.3)	0.702	0.780
Habitual WR before competition (kg)	median (IQR)	1.0 (1.0-2.0)	1.0 (1.0-2.0)	1.0 (1.0-2.0)	—	—
Habitual WR before competition (%)	median (IQR)	2.0 (1.4-3.7)	2.1 (1.4-3.5)	2.0 (1.4-3.7)	0.695	0.780
Habitual WR/WL before competition (%)	median (IQR)	25.0 (16.3-50.0)	20.0 (14.3-42.5)	25.0 (20.0-66.7)	0.014*	0.095
Habitual WR after competition (kg)	median (IQR)	3.0 (2.0-5.0)	3.0 (3.0-5.0)	3.0 (2.0-5.0)	—	—
Habitual WR after competition (%)	median (IQR)	6.3 (4.2-8.9)	6.6 (4.4-8.8)	6.1 (3.8-8.9)	0.435	0.725
Number of WL in the last year	median (IQR)	1 (1-2)	1 (1-2)	1 (1-2)	0.962	0.962

Note: * = significant differences between groups (P < 0.05). — = not available. WL, weight loss. WR, weight regain. IQR, interquartile range. SD, standard deviation.

Among the 125 athletes who reported engaging in WL, no statistically significant differences were observed between medalists and non-medalists after FDR adjustment for any of the WL characteristics assessed in Table 2. In the unadjusted analyses, medalists reported a significantly greater habitual WL% (mean difference = 1.5%, 95% CI: 0.05% to 2.86%, *p* = 0.043, *d* = 0.38) and habitual WL% during the 9–60 days prior to weigh-in (*p* = 0.019, *r*_*rb*_ = 0.25), as well as a lower habitual WR relative to WL (WR/WL) before competition (*p* = 0.014, *r*_*rb*_ = −0.26), compared with non-medalists. The remaining WL characteristics, including age began WL, highest WL%, habitual WL% during 2–8 days prior to weigh-in, habitual WL% during 1 days prior to weigh-in, habitual WR% before competition, habitual WR% after competition, and number of WL in the last year also showed no statistically significant between-group differences in the unadjusted analyses (all *p* > 0.05).

Regarding categorical variables, no significant differences were found between medalists and non-medalists according to sex, sources of WL guidance, or influencing factors (all *p* > 0.05). Similarly, the use of specific WL methods did not differ significantly between medalists and non-medalists (all *p* > 0.05). No significant differences were found between groups in the perception of the impact of WL on health, performance, or fairness (all *p* > 0.05). After FDR adjustment, none of the WL methods or sources of influence showed a statistically significant between-group difference.

Participants mostly allocated 15 + days before the competition for WL (69%), followed by 11–14 days (14%), 8–10 days (8%), 6–7 days (4%), 4–5 days (3%), and 1–3 days (2%). Coaches (78%) were most frequently identified as the primary guides for WL, followed by self-guidance (46%), strength and conditioning coaches (9%), doctors (5%), parents (2%), nutritionists (2%), and other sources (1%). The primary reason reported for engaging in WL was to compete against lighter opponents to increase the chances of winning (74%), followed by optimizing athletic performance (62%), being above usual weight before a competition (40%), others (3%), “because everyone else is cutting weight, so I have to do it too” (1%), and “my coach told me to lose weight, so I have to do it” (1%). Nearly half of participants perceived that WL had no impact on health (47%), whereas 34% perceived it as detrimental and 18% as beneficial. The largest proportion of participants perceived WL as beneficial to performance (39%), followed by 36% who reported no impact and 25% who considered it detrimental. Regarding fairness, most participants indicated that WL did not lead to unfair competition (88%), whereas 11% were uncertain and 1% perceived it as unfair. The WL methods used by athletes are shown in Table 3. The reported influence of different sources on the athletes’ WL practices is shown in Table 4.

**Table 3:** Frequency analysis (n and %) of the weight loss methods used by Chinese adolescent national level Sanda athletes (n = 125).

Methods	Group	Always	Sometimes	Almost never	Never used	Do not use any more	Phi	P	Adjusted P
Gradual dieting	Medalist	10 (8)	10 (8)	11 (9)	15 (12)	0 (0)	0.251	0.095	0.544
	Non-medalist	10 (8)	26 (21)	11 (9)	26 (21)	6 (5)			
Skipping meals	Medalist	11 (9)	14 (11)	10 (8)	10 (8)	1 (1)	0.220	0.197	0.837
	Non-medalist	10 (8)	37 (30)	10 (8)	21 (17)	1 (1)			
Fasting	Medalist	1 (1)	5 (4)	10 (8)	30 (24)	0 (0)	0.156	0.557	0.888
	Non-medalist	2 (2)	3 (2)	20 (16)	53 (42)	1 (1)			
Restricting fluid ingestion	Medalist	12 (10)	16 (13)	7 (6)	11 (9)	0 (0)	0.269	0.060	0.544
	Non-medalist	9 (7)	19 (15)	15 (12)	35 (28)	1 (1)			
Increased exercise	Medalist	27 (22)	13 (10)	1 (1)	4 (3)	1 (1)	0.191	0.333	0.880
	Non-medalist	44 (35)	22 (18)	8 (6)	5 (4)	0 (0)			
Training in a heated room	Medalist	14 (11)	7 (6)	7 (6)	17 (14)	1 (1)	0.176	0.427	0.880
	Non-medalist	13 (10)	14 (11)	18 (14)	31 (25)	3 (2)			
Sauna	Medalist	3 (2)	6 (5)	7 (6)	29 (23)	1 (1)	0.129	0.737	0.895
	Non-medalist	3 (2)	14 (11)	8 (6)	50 (40)	4 (3)			
Training in plastic suits	Medalist	21 (17)	10 (8)	8 (6)	6 (5)	1 (1)	0.187	0.356	0.880
	Non-medalist	31 (25)	27 (22)	9 (7)	12 (10)	0 (0)			
Use plastic suit all-day	Medalist	3 (2)	4 (3)	11 (9)	27 (22)	1 (1)	0.063	0.990	0.990
	Non-medalist	5 (4)	5 (4)	18 (14)	48 (38)	3 (2)			
Spitting	Medalist	3 (2)	8 (6)	8 (6)	26 (21)	1 (1)	0.174	0.466	0.880
	Non-medalist	5 (4)	13 (10)	6 (5)	50 (40)	5 (4)			
Laxatives	Medalist	2 (2)	4 (3)	1 (1)	38 (30)	1 (1)	0.116	0.854	0.968
	Non-medalist	1 (1)	5 (4)	2 (2)	68 (54)	3 (2)			
Diuretics	Medalist	0 (0)	0 (0)	0 (0)	45 (36)	1 (1)	0.129	0.588	0.888
	Non-medalist	0 (0)	0 (0)	3 (2)	73 (58)	3 (2)			
Diet pills	Medalist	1 (1)	1 (1)	0 (0)	43 (34)	1 (1)	0.165	0.662	0.888
	Non-medalist	0 (0)	3 (2)	2 (1)	71 (57)	3 (2)			
Vomiting	Medalist	4 (3)	6 (5)	4 (3)	31 (25)	1 (1)	0.081	0.979	0.990
	Non-medalist	7 (6)	11 (9)	8 (6)	49 (39)	4 (3)			
Hot water immersion	Medalist	1 (1)	2 (2)	5 (4)	38 (30)	0 (0)	0.254	0.096	0.544
	Non-medalist	6 (5)	12 (10)	5 (4)	53 (42)	3 (2)			
Hot saltwater immersion	Medalist	1 (1)	0 (0)	3 (2)	41 (33)	1 (1)	0.193	0.386	0.880
	Non-medalist	0 (0)	3 (2)	9 (7)	64 (51)	3 (2)			
Others	Medalist	3 (2)	0 (0)	2 (2)	39 (31)	2 (2)	0.122	0.679	0.888
	Non-medalist	7 (6)	0 (0)	1 (1)	65 (52)	6 (5)			

Note: The combined percentage of medalists and non-medalists amounts to 100%. Due to rounding rules, some totals do not exactly equal 100%.

**Table 4:** Frequency analysis (n and %) of the sources of influence on the weight loss practices of Chinese adolescent national level Sanda athletes (n = 125).

Sources	Group	Very influential	Some influence	Unsure	Little influence	Not influential	Phi	P	Adjusted P
Other athletes (different sports)	Medalist	1 (1)	2 (2)	5 (4)	1 (1)	37 (30)	0.171	0.529	0.766
	Non-medalist	4 (3)	7 (6)	6 (5)	6 (5)	56 (45)			
Other athletes (same sport)	Medalist	2 (2)	18 (14)	4 (3)	4 (3)	18 (14)	0.142	0.653	0.766
	Non-medalist	5 (4)	24 (19)	4 (3)	12 (10)	34 (27)			
Doctors	Medalist	1 (1)	3 (2)	5 (4)	5 (4)	32 (26)	0.150	0.648	0.766
	Non-medalist	3 (2)	7 (6)	3 (2)	10 (8)	56 (45)			
S&C coaches /physical trainer	Medalist	3 (2)	7 (6)	3 (2)	8 (6)	25 (20)	0.146	0.614	0.766
	Non-medalist	12 (10)	14 (11)	6 (5)	12 (10)	35 (28)			
Coaches	Medalist	21 (17)	10 (8)	1 (1)	4 (3)	10 (8)	0.177	0.418	0.766
	Non-medalist	25 (20)	24 (19)	5 (4)	10 (8)	15 (12)			
Parents	Medalist	10 (8)	13 (10)	1 (1)	8 (6)	14 (11)	0.144	0.626	0.766
	Non-medalist	16 (13)	18 (14)	7 (6)	16 (13)	22 (18)			
Nutritionists	Medalist	1 (1)	2 (2)	6 (5)	2 (2)	35 (28)	0.186	0.364	0.766
	Non-medalist	1 (1)	5 (4)	4 (3)	9 (7)	60 (48)			
Journal articles	Medalist	1 (1)	1 (1)	3 (2)	2 (2)	39 (31)	0.135	0.758	0.766
	Non-medalist	4 (3)	4 (3)	3 (2)	6 (5)	62 (50)			
Book/magazines	Medalist	1 (1)	3 (2)	2 (2)	3 (2)	37 (30)	0.120	0.766	0.766
	Non-medalist	5 (4)	3 (2)	2 (2)	5 (4)	64 (51)			
Internet sources	Medalist	0 (0)	5 (4)	4 (3)	4 (3)	33 (26)	0.190	0.360	0.766
	Non-medalist	5 (4)	6 (5)	3 (2)	7 (6)	58 (46)			
Others	Medalist	0 (0)	1 (1)	2 (2)	1 (1)	42 (34)	0.207	0.275	0.766
	Non-medalist	3 (2)	1 (1)	0 (0)	2 (2)	73 (58)			

Note: S&C, strength and conditioning; Due to rounding rules, some total combined percentage of medalists and non-medalists do not exactly equal 100%.

## Discussion

The present study aimed to compare WL practices and perceptions between medalists and non-medalists among Chinese national-level adolescent Sanda athletes. The findings indicated that these measures were broadly similar between groups, and no statistically significant between-group differences remained after Benjamini–Hochberg FDR adjustment. Although three nominally significant associations were identified in the unadjusted analyses—higher habitual WL%, higher habitual WL% during the 9–60 days prior to weigh-in, and lower habitual WR/WL among medalists—none remained statistically significant after FDR adjustment. These unadjusted associations should therefore be considered exploratory and hypothesis-generating rather than evidence of robust differences in WL characteristics between athletes with and without a history of national-level medal achievement. Overall, the present findings suggest that the WL practices and perceptions assessed in this study did not clearly distinguish national-level adolescent Sanda athletes with and without a history of national medal achievement.

In this study, 79% of athletes reported engaging in purposeful WL, which is higher than most previous findings in adolescent CS athletes (57–73%) [6,7,9,10,14,15,18,22,32,34,36], and comparable only to adolescent judo athletes (80%) [25]. This relatively high prevalence is also similar to that reported in adult CS athletes and may be related to the national-level competitive context of the present sample [4]. At the national level, where athletes compete within closely matched weight categories and performance levels, WL may be commonly used to meet weight-class requirements while pursuing potential competitive advantages [10]. The high prevalence observed in the present study therefore suggests that intentional WL may represent a common component of competitive preparation among national-level adolescent Sanda athletes, regardless of medal achievement. This finding highlights the importance of addressing WL practices at the population level rather than focusing only on athletes with greater competitive achievement.

Although most WL practices were comparable between medalists and non-medalists (Tables 2 and 3), three nominally significant between-group associations were identified in the unadjusted analyses: habitual WL%, habitual WL% during the 9–60 days prior to weigh-in, and habitual WR/WL before competition. However, none of these differences remained statistically significant after Benjamini–Hochberg FDR adjustment. Thus, the observed unadjusted differences should be interpreted cautiously as exploratory findings rather than robust differences between medalists and non-medalists. The higher habitual WL% and higher habitual WL% during the 9–60 days prior to weigh-in observed among medalists in the unadjusted analyses may warrant further investigation, but the present data do not provide robust evidence that these WL variables differ according to medal achievement. Similarly, the lower habitual WR/WL before competition observed among medalists should not be interpreted as evidence of a distinct or superior approach to post-weigh-in weight recovery.

More broadly, no statistically significant differences were observed between medalists and non-medalists in the sources of guidance, influencing factors, WL methods, or perceptions of WL, including health, performance, and fairness. The overall consistency across these domains may partly reflect the shared competitive and training environment experienced by national-level adolescent Sanda athletes. Athletes competing at this level may be exposed to similar weight-class requirements, coaching practices, training routines, and competition norms, which may result in considerable similarity in how WL is understood and practiced. Such a relatively homogeneous environment may reduce the extent to which WL practices distinguish athletes according to competitive achievement. At the same time, competitive achievement is likely influenced by multiple interacting factors, whereas the present study examined only WL practices and perceptions. Factors such as technical and tactical proficiency, physical fitness, training quality, competitive experience, and coaching support were not assessed in the present study and may also contribute to differences in competitive achievement. Therefore, the absence of statistically significant between-group differences across these WL-related domains suggests that the WL characteristics assessed in this study were not sufficient to distinguish athletes with and without a history of national-level medal achievement.

Beyond the comparison of medal achievement, the magnitude of WL reported by the athletes warrants attention. In the present study, athletes who engaged in WL reported a relatively substantial habitual WL magnitude (8.7 ± 3.9% of off-season BM), while the median highest WL was 11.3% of BM. Previous studies have indicated that WL exceeding 5% of BM may negatively affect physical and psychological health [59,60]. This is particularly relevant for adolescent athletes, who are undergoing ongoing growth and development and may therefore be more vulnerable to the consequences of repeated or substantial energy and fluid restriction. Although distributing WL over a longer period may reduce some of the acute physiological stresses associated with rapid short-term WL [61–63], this does not necessarily eliminate the potential risks associated with a large overall magnitude or repeated episodes of WL. In particular, substantial cumulative BM loss may have implications for energy availability, growth, bone health, and overall physical development [52–55]. Importantly, these concerns apply to the population as a whole and are not contingent on medal achievement. Therefore, the high prevalence and substantial magnitude of WL observed in this population warrant attention as a broader athlete-health issue among national-level adolescent Sanda athletes, rather than only as a characteristic potentially related to competitive achievement.

Several study limitations should be acknowledged. First, the cross-sectional design precludes causal inference regarding the relationship between WL practices and competitive success. Moreover, medalist status was defined according to whether an athlete had achieved a national-level medal at any point in their competitive career, whereas WL characteristics were based on retrospectively reported habitual practices. Therefore, the temporal relationship between reported WL practices and medal achievement could not be established. Second, the use of self-reported questionnaire data may introduce recall bias and reporting inaccuracies. Furthermore, because participants were adolescents undergoing growth and development, natural fluctuations in body mass during the 60-day pre-competition period may have affected the accuracy of the reported habitual WL. Third, although the sample was restricted to national-level adolescent Sanda athletes to to provide a relatively homogeneous competitive population, these findings may not be generalizable to other populations, age groups, or CS with different competition structures [64]. Fourth, the relatively modest sample size and imbalance in some participant characteristics may have limited the ability to detect small between-group differences, particularly for subgroup or sex-specific analyses. Finally, because the medalist comparisons were unadjusted, residual confounding by sex, age, competitive experience, and other factors cannot be excluded. The relatively small number of female athletes limited the feasibility of sex-stratified analyses without compromising statistical power. Therefore, the findings should be interpreted with caution, and future studies with larger and more balanced samples and prospective designs are needed to further examine potential sex-specific differences in WL practices.

## Conclusion

In conclusion, WL practices and perceptions were similar between medalists and non-medalists among Chinese national-level adolescent Sanda athletes. Although three nominal associations were identified in the unadjusted analyses, none remained statistically significant after Benjamini–Hochberg FDR adjustment. Therefore, the present findings do not provide robust evidence of systematic differences in WL practices or perceptions according to a history of national-level medal achievement. The high prevalence and substantial magnitude of WL observed in this population nevertheless warrant attention, particularly given the developmental stage of adolescent athletes. The nominal associations identified in the unadjusted analyses may serve as hypothesis-generating findings for future longitudinal studies, but should not be interpreted as evidence of a superior, safer, or performance-enhancing WL pattern associated with competitive success.

## Supporting information

S1 File STROBE-checklist. (DOCX)
