# Supplementary material for: Differences in weight loss practices and perceptions between medalists and non-medalists in national-level adolescent Sanda athletes
Source: PLoS One. 2026 Sep 24;21(9):e0359088. doi: 10.1371/journal.pone.0359088 (PMC13606930; doi:10.1371/journal.pone.0359088)
Supplement: Supplementary file 2 [file pone.0359088.s002.docx]

**(Manuscript ID: PONE-D-26-30122)**

**Dear Editor and Reviewers:**

Thank you for your positive response and for providing the reviewers’ comments concerning our manuscript entitled “Differences in weight loss strategies between medalists and non-medalists in national-level adolescent Sanda athletes”.

The reviewer’s comments have significantly improved our paper. We highlighted the changes of the manuscript within the document by tracked change.

**Response to Reviewer 1:**

**Reviewer:**

1. Statistical Significance vs. Practical Meaningfulness (Missing Effect Sizes)

The Issue: In Table 2, you report a statistically significant difference in habitual WL% between medalists (9.6 ± 3.5%) and non-medalists (8.1 ± 4.0%) with a p-value of 0.043. The mean difference is a mere 1.5%, and the 95% confidence interval (0.05% to 2.86%) barely scratches the surface of zero. For a 60kg adolescent athlete, a 1.5% difference equates to roughly 900g.

The Critique: Relying solely on a fragile p-value (p = 0.043) without reporting effect sizes (e.g., Cohen’s d or Hedges’ g) is insufficient for a journal of PLOS ONE’s caliber. A 900g absolute difference in habitual weight cutting across months cannot be logically or biochemically crowned as a "distinct strategic pattern" or a "decisive model for competitive success".

Action Required: Report the exact effect sizes for all significant continuous variables (habitual WL%, early phase WL%, and WR/WL). The discussion must be toned down to reflect that the actual magnitude of difference between the two groups is marginal at best.

**Reply:** We appreciate the reviewer's thoughtful comment regarding the distinction between statistical significance and practical significance. Following this suggestion, we have revised the manuscript in several ways.

First, we now report effect sizes for all statistically significant continuous variables. Specifically, Cohen's d was reported for the normally distributed variable (habitual WL%), whereas rank-biserial correlation (r<sub>rb</sub>) was reported for variables analyzed using the Mann–Whitney U test. These effect sizes indicated that the observed between-group differences were small. We agree that statistical significance should be interpreted together with effect size and clinical/practical relevance rather than P values alone.

Second, we have revised the Discussion to explicitly acknowledge the modest magnitude of the observed differences. Statements describing medalists as exhibiting a "distinct strategic pattern" or implying a "decisive model for competitive success" have been removed or substantially toned down. The revised manuscript now emphasizes that only three modest differences were observed, that the corresponding effect sizes were small, and that these findings should not be interpreted as evidence of a superior or causal WL strategy.

Finally, the Abstract and Conclusion have also been revised to ensure that the interpretation of the findings is appropriately cautious and reflects the limited magnitude of the observed differences.

**Reviewer:**

2. Flawed Causal Inference from Cross-Sectional Design

The Issue: The conclusion states that "competitive differences may be associated not with the specific WL methods used, but with how WL is structured and regulated over time," framing a structured WL strategy as a driver/cause of winning a medal.

The Critique: This is a classic cross-sectional study using purposive sampling. You cannot infer directionality. It is highly plausible that medalists are simply more experienced, have competed longer, or belong to better-funded elite training bases where coaches enforce stricter monitoring, which consequently leads to better weight management. The "strategy" could be a byproduct of being an elite medalist, not the cause of it.

Action Required: Reword the Abstract, Discussion, and Conclusion to completely eliminate any causal or predictive undertones. Explicitly acknowledge the limitation of reverse causality in the limitations section.

**Reply:** We appreciate the reviewer's important comment regarding causal inference. We agree that the cross-sectional design of the present study does not permit conclusions regarding the direction of the observed associations.

Accordingly, we have revised the Abstract, Discussion, and Conclusion to remove causal or predictive language throughout the manuscript. Expressions implying that habitual WL strategies may "drive" or explain competitive success have been replaced with more neutral descriptions emphasizing observed between-group differences only.

In addition, we have expanded the limitations section to explicitly acknowledge the possibility of reverse causality. Specifically, we now state that the observed differences may reflect greater competitive experience or other unmeasured factors associated with successful athletes, rather than habitual WL practices contributing to competitive success.

**Reviewer:**

3. The "Exploratory Nature" Excuse and Type I Error Inflation

The Issue: In the Statistical Analyses section, the authors state that "given the exploratory nature of the study and the absence of a single primary outcome, no formal adjustment for multiple comparisons was applied."

The Critique: You ran dozens of independent t-tests, Mann-Whitney U tests, and Chi-square tests across Tables 1, 2, 3, and 4. When conducting dozens of simultaneous hypotheses tests at alpha = 0.05, the family-wise error rate (the probability of falsely detecting at least one significant difference due to sheer randomness) inflates exponentially. Your borderline p-values (p = 0.043 for habitual WL%) are highly susceptible to being false positives (Type I error).

Action Required: Apply a formal correction for multiple comparisons (e.g., Benjamini-Hochberg False Discovery Rate or Bonferroni) to verify if these differences survive. If they do not, the core narrative of the paper must change.

**Reply:** We appreciate the reviewer's concern regarding inflation of Type I error. We acknowledge that multiple statistical comparisons increase the probability of false-positive findings.

However, the present study was designed as an exploratory cross-sectional investigation without a single predefined primary outcome. Rather than testing multiple hypotheses for one endpoint, the analyses examined different domains of habitual WL practices (Age began WL, Highest WL (%), Habitual WL (%), Habitual WL during 9-60, 2-8, 1 days prior to weigh-in (%), habitual WR before competition (%), habitual WL/WR, habitual WR after competition (%), Number of WL in the last year), specific methods, sources of influence, and perceptions. Therefore, we did not apply a formal family-wise error correction, as such adjustments may be overly conservative in exploratory observational research and substantially increase the risk of Type II error.

Instead, we have strengthened the manuscript by (i) reporting effect sizes for all statistically significant continuous variables, (ii) substantially moderating the interpretation of the findings throughout the Abstract, Discussion, and Conclusion, and (iii) explicitly acknowledging in the Limitations that no formal adjustment for multiple comparisons was performed and that the statistically significant findings should be regarded as exploratory and confirmed in future studies.

**Reviewer:**

4. Methodological Disconnect in the "Multi-Day Competition" Logic

The Issue: In the Discussion, the authors argue that lower habitual WR/WL in medalists reflects a controlled approach tailored to the demands of "multi-day Sanda competitions" where body mass stability across consecutive days is crucial.

The Critique: This is a massive leap in logic. Your questionnaire captured habitual weight regain relative to weight loss before the competition cycle (pre-weigh-in phase). It does not capture the actual day-to-day weight fluctuations or acute post-weigh-in fluid/energy titration during a multi-day tournament. You are using pre-competition recall data to justify an in-competition physiological strategy.

Action Required: Either remove the speculative multi-day competition hypothesis or clearly state that the study lacked the granulatory data (such as daily weigh-in charts during tournaments) to substantiate this claim.

**Reply:** Thank you for this insightful comment. We agree that our original discussion overstated the implications of the observed WR/WL difference for body mass regulation during multi-day competitions. We acknowledge that the study did not collect repeated day-to-day body mass measurements during competitions. Therefore, the present data cannot directly support inferences regarding body mass fluctuations or recovery dynamics across consecutive competition days. Accordingly, we have revised this section of the Discussion to focus on the observed difference in habitual WR relative to WL, removed the stronger interpretation regarding multi-day competition physiology, and explicitly state that any explanation for this finding remains speculative and requires confirmation in future longitudinal studies..

**Reviewer:**

5. Recall Bias and Developmental Vulnerability of Adolescents

The Issue: The study heavily relies on retrospective self-reporting via the RWLQ in a very young cohort (12-17 years old).

The Critique: Asking a 14-year-old athlete to recall their exact "habitual weight loss percentage during the 9-60 days prior to weigh-in" introduces massive recall bias. Sixty days prior is a massive window for an adolescent undergoing rapid somatic growth and unpredictable growth spurts.

Action Required: The authors must discuss how developmental growth fluctuations over a 60-day window confound the "intentional" WL% reported by these young athletes.

**Reply:** Thank you for this valuable comment. We agree that retrospective self-report may introduce recall bias, particularly in adolescent athletes. However, we would like to clarify that participants were not asked to recall exact weight loss percentages. Instead, the questionnaire asked athletes to report the amount of body mass (kg) they typically lost and regained before competition, which is a familiar aspect of their routine weight management. Weight loss percentages were subsequently calculated by the researchers based on the reported values. Nevertheless, we acknowledge that natural growth and developmental changes during adolescence, together with the retrospective nature of the questionnaire, may have influenced the estimation of habitual WL over the 60-day pre-competition period. This limitation has now been added to the Discussion.

**Reviewer:**

6. Table 2 Discrepancy: The row label says habitual WL/WR before competition (%) but the abstract and results text reference lower habitual weight regain relative to WL (WR/WL). Please standardize whether you are calculating WR divided by WL, or WL divided by WR, as the table label currently contradicts the text.

**Reply:** Thank you for your comment. We revised it.

**Reviewer:**

7. Table 3 and 4 Power: Given that the medalist group (n=46) and non-medalist group (n=79) have an uneven distribution, and many cells in the Chi-square/Phi analysis have frequencies < 5 (e.g., Diuretics, Laxatives, Diet pills), a standard Chi-square test is violated. Fisher’s Exact Test should be utilized and stated for these low-frequency categorical variables.

**Reply:** Thank you for this helpful comment. We agree that Pearson's chi-square test may not be appropriate for contingency tables with sparse expected cell frequencies. Accordingly, categorical variables that violated the assumptions of Pearson's chi-square test were reanalyzed using Fisher–Freeman–Halton exact tests. The Methods section has been revised accordingly. The exact tests produced results consistent with the original analyses.

**Again, we would like to thank you again for reviewers’ comments. Your valuable opinions have improved the quality of the manuscript. We hope we have satisfactorily addressed the comments that were made.**

**Response to Reviewer 2:**

**Reviewer:**

154-156. The justification for not performing an a priori sample size calculation is acceptable for an exploratory study involving a limited population. However, the manuscript does not clearly describe the sampling strategy. Please specify whether all eligible athletes were invited to participate or whether a convenience sample was used. The current wording ("all available athletes accessible") is ambiguous and should not be interpreted as implying random or representative sampling. Being a hard-to-reach population does not justify the absence of an a priori sample size calculation.

***Reply***: Thank you for this valuable comment. We agree that the original description of the sampling strategy was insufficiently clear. The Methods section has been revised to explicitly state that purposive and convenience sampling were used, and that all athletes meeting the inclusion criteria who were accessible through the participating sports teams during the recruitment period were invited to participate. We also revised the statement regarding the absence of an a priori sample size calculation to avoid implying that the hard-to-reach nature of the population justified this decision. Instead, we clarify that recruitment continued throughout the predefined data collection period to maximize participation in this exploratory cross-sectional study.

**Reviewer:**

172. What is the scientific validity of the RWLQ developed by Zhong et al.? Specifically, what are its psychometric properties (e.g., Cronbach's alpha), and what response format or rating scale does it use?

***Reply***: Thank you for this helpful comment. We apologize for the ambiguity in the original manuscript. The questionnaire used in this study was not newly developed by Zhong et al., but was an adapted version of the Rapid Weight Loss Questionnaire (RWLQ) originally developed and validated by Artioli et al (ICC > 0.90 for all questions whose answers were parametric, Cronbach’s a was 0.98 for scores obtained between test and retest). Zhong et al. subsequently adapted the questionnaire for Chinese Sanda athletes by adding several descriptive items while retaining the original questionnaire structure. We have revised the Methods section to clarify the origin of the questionnaire and the nature of the adaptation.

The original RWLQ has established psychometric validity, whereas the adaptations introduced by Zhong et al. consisted of additional descriptive questions rather than modifications to the validated measurement structure. We have also clarified the questionnaire structure and response formats in the Methods section to improve transparency.

Reference: Artioli, G.G., Scagliusi, F., Kashiwagura, D., Franchini, E., Gualano, B. and Junior, A.L. (2010), Development, validity and reliability of a questionnaire designed to evaluate rapid weight loss patterns in judo players. Scandinavian Journal of Medicine & Science in Sports, 20: e177-e187. https://doi.org/10.1111/j.1600-0838.2009.00940.x

**Reviewer:**

185. For which variable was the t-test applied, given that almost all variables did not meet the assumption of normality?

***Reply***: Thank you for this helpful comment. We agree that the original description of the statistical analyses did not clearly indicate which variable satisfied the assumption of normality. We have revised the Statistical Analysis section to explicitly state that only habitual WL (%) was normally distributed and was therefore analyzed using an independent-samples t-test, whereas all other continuous variables were analyzed using the Mann–Whitney U test.

**Reviewer:**

Table 1- Table 2. In the tables, the mean and standard deviation should not be reported for variables showing a highly skewed distribution, as the standard deviation values exceed the mean, indicating that these statistics may provide a misleading description of the data. Therefore, the median and interquartile range (IQR) should be reported, as they are considered more appropriate indicators of central tendency and dispersion.

***Reply***: Thank you for this valuable suggestion. We agree that mean ± SD is not appropriate for variables with skewed distributions. Accordingly, we have revised Tables 1 and 2 so that normally distributed continuous variables are presented as mean ± SD, whereas non-normally distributed continuous variables are presented as median (IQR). This presentation is now consistent with the distribution of each variable and the statistical methods described in the revised Methods section.

**Again, we would like to thank you again for comments, which improves the quality of the manuscript.**
