# Supplementary material for: Differences in weight loss practices and perceptions between medalists and non-medalists in national-level adolescent Sanda athletes
Source: PLoS One. 2026 Sep 24;21(9):e0359088. doi: 10.1371/journal.pone.0359088 (PMC13606930; doi:10.1371/journal.pone.0359088)
Supplement: Supplementary file 3 [file pone.0359088.s003.docx]

**(Manuscript ID: PONE-D-26-30122)**

**Dear Editor and Reviewers:**

Thank you for providing the reviewers’ comments concerning our manuscript entitled “Differences in weight loss strategies between medalists and non-medalists in national-level adolescent Sanda athletes”.

The reviewer’s comments have significantly improved our paper. We highlighted the changes of the manuscript within the document by tracked change (yellow background).

**Response to Reviewer 1:**

**Reviewer:**

Major Concern 1. The statistical analysis remains insufficiently rigorous

The manuscript examines a large number of weight-loss-related outcomes, including the timing and magnitude of weight loss, weight regain, ratios, frequency, methods, sources of influence, and perceptions. Nevertheless, the analysis appears to rely predominantly on multiple unadjusted group comparisons, with no appropriate control for the substantial multiplicity of statistical testing.

The authors justify the absence of multiplicity adjustment by describing the study as exploratory. However, an exploratory designation does not eliminate the risk of inflated type I error. This is particularly important because the statistically significant findings appear to be relatively few and are associated with modest effect sizes and p-values close to the conventional significance threshold. Consequently, it is difficult to determine whether the reported significant findings represent robust associations or false-positive findings arising from the large number of comparisons.

At minimum, the authors should clearly distinguish prespecified primary outcomes from exploratory analyses and provide an appropriate sensitivity analysis for multiple testing. Without such an analysis, the statistical evidence for the manuscript's principal claims remains weak.

**Reply:** Thank you for this important comment. We agree that the large number of statistical comparisons increases the risk of inflated type I error. We therefore conducted a Benjamini–Hochberg FDR sensitivity analysis, with related outcomes grouped into four conceptual outcome families. The study was exploratory in nature and did not prespecify a single primary WL outcome.

None of the three nominally significant associations identified in the original unadjusted analyses (habitual WL%, habitual WL% during the 9–60 days prior to weigh-in, and habitual WR/WL before competition) remained statistically significant after FDR adjustment. We have therefore revised the Abstract, Results, Discussion, and Conclusion to clearly distinguish these unadjusted findings as exploratory and hypothesis-generating, rather than robust between-group differences. The revised manuscript now emphasizes that the available data do not provide evidence of systematic differences in WL practices or perceptions according to medal achievement.

**Reviewer:**

Major Concern 2. Potential confounding has not been adequately addressed

The central comparison is between medalists and non-medalists, yet the manuscript does not adequately account for variables that could plausibly influence both competitive achievement and weight-loss practices. Relevant factors may include age, sex, competitive experience, number of competitions, training environment, and other characteristics related to competitive exposure.

The decision not to adjust for sex because the medalist and non-medalist groups did not differ statistically in sex distribution is not a sufficient methodological justification. The absence of a statistically significant baseline group difference does not establish that a variable cannot confound an association.

More importantly, the manuscript reports that competition-related characteristics are associated with weight-loss practices, yet these factors are not incorporated into an adjusted analysis of medalist status. Therefore, the observed differences cannot confidently be attributed to medalist status itself.

The authors should either conduct appropriately adjusted analyses or substantially reduce the strength of the conclusions and explicitly frame the findings as unadjusted exploratory associations.

**Reply:** Thank you for this important comment. We agree that potential confounding should be considered when comparing medalists and non-medalists. In response, we substantially reduced the strength of the interpretation rather than attributing the observed unadjusted associations to medalist status.

Importantly, after the Benjamini–Hochberg FDR sensitivity analysis, none of the originally observed WL-related associations remained statistically significant. Accordingly, the revised manuscript no longer presents medalist status as being associated with specific WL characteristics, and all unadjusted findings are explicitly described as nominal, exploratory, and hypothesis-generating. We therefore did not perform a post hoc multivariable adjustment analysis, as there was no longer a statistically significant association that we sought to model as an independent effect of medalist status. Nevertheless, we acknowledge that residual confounding by sex, age, competitive experience, number of competitions, and other unmeasured factors cannot be excluded and have added this limitation to the revised manuscript.

**Reviewer:**

Major Concern 3. The temporal relationship between medal achievement and reported weight-loss behavior is unclear

The manuscript defines medalists based on whether an athlete had achieved a national-level medal during their career, whereas the weight-loss variables represent retrospectively reported habitual practices. This creates an important temporal ambiguity.

It is unclear whether the reported weight-loss practices occurred before or after the athlete's medal achievement. Therefore, the data cannot establish that a particular weight-loss strategy contributed to competitive success. Conversely, the possibility that competitive experience or achievement influenced subsequent weight-loss behavior cannot be excluded.

Accordingly, statements suggesting that medalists "adopt" particular strategies or that specific weight-loss patterns may contribute to competitive success are not adequately supported by the study design.

The conclusions should be restricted to associations between reported weight-loss characteristics and a history of medal achievement.

**Reply:** Thank you for this important comment. We agree that the cross-sectional design and retrospective assessment of WL practices do not allow us to establish the temporal sequence between WL behavior and medal achievement. We have substantially revised the interpretation and conclusions throughout the manuscript and removed statements suggesting that medalists adopted particular WL strategies or that specific WL patterns contributed to competitive success. We also acknowledged this limitation in the limitation section.

**Reviewer:**

Major Concern 4. Several interpretations extend beyond what was actually measured

The manuscript interprets the greater proportion of weight loss occurring during the earlier 9–60-day period among medalists as evidence of a "more gradual" or better-organized weight-loss strategy. However, the questionnaire does not directly establish that these athletes followed a controlled or physiologically safer gradual weight-loss protocol.

Similarly, the lower WR/WL ratio among medalists should not be interpreted as evidence of "tighter control" of post-weigh-in recovery. The reported difference in the ratio appears to be driven substantially by the denominator, because absolute weight regain itself was not significantly different between groups.

The discussion should therefore distinguish carefully between what was directly measured and what is inferred from those measurements.

**Reply:** Thank you for this important comment. We agree that these interpretations extended beyond what was directly measured by the questionnaire. We have therefore removed the descriptions of a “more gradual” or “better-organized” WL strategy and the interpretation of lower WR/WL as “tighter control” of post-weigh-in recovery. We now distinguish more clearly between the measured WL and WR variables and their possible interpretations. In addition, because these associations did not remain statistically significant after FDR adjustment, they are now presented only as nominal, exploratory findings rather than evidence of distinct WL or WR strategies.

**Reviewer:**

Major Concern 5. Claims regarding multi-day competition physiology are not sufficiently supported by the collected data

The manuscript discusses multi-day Sanda competition and suggests that limiting post-weigh-in weight regain may contribute to smaller body-mass fluctuations and more stable physiological conditions. However, the present study did not directly measure day-to-day body-mass fluctuations, hydration status, physiological stability, performance, or recovery during competition.

These statements therefore go beyond the empirical data presented in this study. They may be reasonable hypotheses for future research, but they should not be presented as interpretations supported by the current dataset.

**Reply:** Thank you for your comment. Regarding this part, we have already removed it in the previous round.

**Reviewer:**

Major Concern 6. The practical implications are stronger than warranted by the observational design

The manuscript suggests that earlier and more gradual weight loss may be beneficial and implies that the temporal organization of weight loss may be an important practical target.

However, this is a retrospective observational study without a direct performance outcome or prospective assessment of health consequences. The finding that medalists reported a different temporal distribution of weight loss does not demonstrate that earlier or more gradual weight loss improves performance, health, or competitive success.

The practical implications should therefore be substantially moderated. The data support the identification of an association or behavioral pattern, not a recommendation that one weight-loss strategy is superior.

**Reply:** Thank you for this important comment. We agree that the original practical implications were too strong for the observational design. We have substantially revised the practical interpretation and removed statements suggesting that earlier or more gradual WL is beneficial or should be recommended as a preferred strategy. Given that the observed WL-related associations did not remain statistically significant after FDR adjustment, the revised manuscript does not propose any WL pattern as superior or performance-enhancing.

**Reviewer:**

Major Concern 7. The manuscript's central terminology does not fully correspond to the evidence

The title and several parts of the manuscript refer to "weight-loss strategies." However, the study does not demonstrate that medalists and non-medalists differed substantially in specific weight-loss methods. Rather, the principal differences appear to concern selected characteristics such as the timing or magnitude of reported weight loss and weight regain.

The terminology should therefore be reconsidered so that the title and conclusions accurately reflect the observational and self-reported nature of the data.

**Reply:**

Thank you for this comment. We agree that the term “weight-loss strategies” could overstate what was measured in this observational, self-reported study. We have therefore revised the terminology throughout the manuscript, including the title, Abstract, Discussion, and Conclusion.

**Reviewer:**

Major Concern 8. The revised manuscript still contains substantial editing and language problems

The revised manuscript contains numerous unresolved track-change remnants, duplicated or contradictory wording, typographical errors, and grammatical problems. Examples include constructions such as "standardized similar among between," "includingNevertheless," "Hhabitual WLWR/WLR," and other incomplete or duplicated phrases.

These issues substantially reduce readability and create ambiguity in several methodological and interpretational statements. Given that PLOS ONE does not provide substantive copyediting after acceptance, the manuscript requires a careful and complete language and formatting revision before it can be considered publication-ready.

**Reply:** Thank you for pointing this out, and we sincerely apologize for the confusion and inconvenience. PLOS ONE requires both a tracked-changes version and a clean version for submission. In the previous submission, the tracked-changes Word file was converted to PDF in a way that retained both the original and revised text, which resulted in duplicated and apparently contradictory wording. We acknowledge that this substantially affected the readability of the manuscript.

In the present revision, we have carefully reviewed the entire manuscript, removed all Track Changes and edited remnants, and corrected grammatical, typographical, and formatting errors. The revised manuscript is presented as a clean version, with changes highlighted in yellow instead of using Track Changes. We have also carefully checked the manuscript to ensure that no duplicated or contradictory text remains.

**Again, we would like to thank you again for reviewers’ comments. Your valuable opinions have improved the quality of the manuscript. We hope we have satisfactorily addressed the comments that were made.**
