# Supplementary material for: Differences in weight loss practices and perceptions between medalists and non-medalists in national-level adolescent Sanda athletes
Source: PLoS One. 2026 Sep 24;21(9):e0359088. doi: 10.1371/journal.pone.0359088 (PMC13606930; doi:10.1371/journal.pone.0359088)
Supplement: Peer review history

# Peer review: Decision Letter 0

**DOI:** 10.1371/journal.pone.0359088.r001

**Authors:** Stefano Amatori

17 Jul 2026

Dear Dr. Zhong,

Thank you for submitting your manuscript to PLOS ONE. After careful consideration, we feel that it has merit but does not fully meet PLOS ONE’s publication criteria as it currently stands. Therefore, we invite you to submit a revised version of the manuscript that addresses the points raised during the review process.

Please submit your revised manuscript by Sep 15 2026 11:59PM. If you will need more time than this to complete your revisions, please reply to this message or contact the journal office at plosone@plos.org. When you're ready to submit your revision, log on to https://www.editorialmanager.com/pone/ and select the 'Submissions Needing Revision' folder to locate your manuscript file.

- A letter that responds to each point raised by the academic editor and reviewer(s). You should upload this letter as a separate file labeled 'Response to Reviewers'.
- A marked-up copy of your manuscript that highlights changes made to the original version. You should upload this as a separate file labeled 'Revised Manuscript with Track Changes'.
- An unmarked version of your revised paper without tracked changes. You should upload this as a separate file labeled 'Manuscript'.

If you would like to make changes to your financial disclosure, please include your updated statement in your cover letter. Guidelines for resubmitting your figure files are available below the reviewer comments at the end of this letter.

If applicable, we recommend that you deposit your laboratory protocols in protocols.io to enhance the reproducibility of your results. Protocols.io assigns your protocol its own identifier (DOI) so that it can be cited independently in the future. For instructions see: https://journals.plos.org/plosone/s/submission-guidelines#loc-laboratory-protocols. Additionally, PLOS ONE offers an option for publishing peer-reviewed Lab Protocol articles, which describe protocols hosted on protocols.io. Read more information on sharing protocols at https://plos.org/protocols?utm_medium=editorial-email&utm_source=authorletters&utm_campaign=protocols.

As the corresponding author, your ORCID iD is verified in the submission system and will appear in the published article. PLOS supports the use of ORCID, and we encourage all coauthors to register for an ORCID iD and use it as well. Please encourage your coauthors to verify their ORCID iD within the submission system before final acceptance, as unverified ORCID iDs will not appear in the published article. *Only*  the individual author can complete the verification step; PLOS staff *cannot*  verify ORCID iDs on behalf of authors.

We look forward to receiving your revised manuscript.

Kind regards,

Stefano Amatori, Ph.D.

Academic Editor

PLOS One

Journal Requirements:

When submitting your revision, we need you to address these additional requirements.

1. Please ensure that your manuscript meets PLOS ONE's style requirements, including those for file naming. The PLOS ONE style templates can be found at

https://journals.plos.org/plosone/s/file?id=wjVg/PLOSOne_formatting_sample_main_body.pdf and https://journals.plos.org/plosone/s/file?id=ba62/PLOSOne_formatting_sample_title_authors_affiliations.pdf

2. Please include your full ethics statement in the ‘Methods’ section of your manuscript file. In your statement, please include the full name of the IRB or ethics committee who approved or waived your study, as well as whether or not you obtained informed written or verbal consent. If consent was waived for your study, please include this information in your statement as well.

3. If the reviewer comments include a recommendation to cite specific previously published works, please review and evaluate these publications to determine whether they are relevant and should be cited. There is no requirement to cite these works unless the editor has indicated otherwise.

[Note: HTML markup is below. Please do not edit.]

Reviewers' comments:

Reviewer's Responses to Questions

**Comments to the Author**

1. Is the manuscript technically sound, and do the data support the conclusions?

Reviewer #1: Yes

Reviewer #2: Yes

**********

2. Has the statistical analysis been performed appropriately and rigorously?

Reviewer #1: No

Reviewer #2: No

**********

3. Have the authors made all data underlying the findings in their manuscript fully available?

The PLOS Data policy

Reviewer #1: No

Reviewer #2: No

**********

4. Is the manuscript presented in an intelligible fashion and written in standard English?

Reviewer #1: Yes

Reviewer #2: Yes

**********

Reviewer #1: General Impression: The manuscript investigated the differences in habitual weight loss (WL) practices and perceptions between medalists and non-medalists among Chinese national-level adolescent Sanda athletes. While mapping the epidemiological landscape of WL in adolescent combat sports is an important and challenging endeavor, the manuscript in its current form suffers from serious methodological limitations, logical fallacies regarding causality, and statistical vulnerabilities that undermine the validity of its conclusions.

Major Comments

1. Statistical Significance vs. Practical Meaningfulness (Missing Effect Sizes)The Issue: In Table 2, you report a statistically significant difference in habitual WL% between medalists (9.6 ± 3.5%) and non-medalists (8.1 ± 4.0%) with a p-value of 0.043. The mean difference is a mere 1.5%, and the 95% confidence interval (0.05% to 2.86%) barely scratches the surface of zero. For a 60kg adolescent athlete, a 1.5% difference equates to roughly 900g.

The Critique: Relying solely on a fragile p-value (p = 0.043) without reporting effect sizes (e.g., Cohen’s d or Hedges’ g) is insufficient for a journal of PLOS ONE’s caliber. A 900g absolute difference in habitual weight cutting across months cannot be logically or biochemically crowned as a "distinct strategic pattern" or a "decisive model for competitive success".

Action Required: Report the exact effect sizes for all significant continuous variables (habitual WL%, early phase WL%, and WR/WL). The discussion must be toned down to reflect that the actual magnitude of difference between the two groups is marginal at best.

2. Flawed Causal Inference from Cross-Sectional Design

The Issue: The conclusion states that "competitive differences may be associated not with the specific WL methods used, but with how WL is structured and regulated over time," framing a structured WL strategy as a driver/cause of winning a medal.

The Critique: This is a classic cross-sectional study using purposive sampling. You cannot infer directionality. It is highly plausible that medalists are simply more experienced, have competed longer, or belong to better-funded elite training bases where coaches enforce stricter monitoring, which consequently leads to better weight management. The "strategy" could be a byproduct of being an elite medalist, not the cause of it.

Action Required: Reword the Abstract, Discussion, and Conclusion to completely eliminate any causal or predictive undertones. Explicitly acknowledge the limitation of reverse causality in the limitations section.

3. The "Exploratory Nature" Excuse and Type I Error Inflation

The Issue: In the Statistical Analyses section, the authors state that "given the exploratory nature of the study and the absence of a single primary outcome, no formal adjustment for multiple comparisons was applied."

The Critique: You ran dozens of independent t-tests, Mann-Whitney U tests, and Chi-square tests across Tables 1, 2, 3, and 4. When conducting dozens of simultaneous hypotheses tests at alpha = 0.05, the family-wise error rate (the probability of falsely detecting at least one significant difference due to sheer randomness) inflates exponentially. Your borderline p-values (p = 0.043 for habitual WL%) are highly susceptible to being false positives (Type I error).

Action Required: Apply a formal correction for multiple comparisons (e.g., Benjamini-Hochberg False Discovery Rate or Bonferroni) to verify if these differences survive. If they do not, the core narrative of the paper must change.

4. Methodological Disconnect in the "Multi-Day Competition" Logic

The Issue: In the Discussion, the authors argue that lower habitual WR/WL in medalists reflects a controlled approach tailored to the demands of "multi-day Sanda competitions" where body mass stability across consecutive days is crucial.

The Critique: This is a massive leap in logic. Your questionnaire captured habitual weight regain relative to weight loss before the competition cycle (pre-weigh-in phase). It does not capture the actual day-to-day weight fluctuations or acute post-weigh-in fluid/energy titration during a multi-day tournament. You are using pre-competition recall data to justify an in-competition physiological strategy.

Action Required: Either remove the speculative multi-day competition hypothesis or clearly state that the study lacked the granulatory data (such as daily weigh-in charts during tournaments) to substantiate this claim.

5. Recall Bias and Developmental Vulnerability of Adolescents

The Issue: The study heavily relies on retrospective self-reporting via the RWLQ in a very young cohort (12-17 years old).

The Critique: Asking a 14-year-old athlete to recall their exact "habitual weight loss percentage during the 9-60 days prior to weigh-in" introduces massive recall bias. Sixty days prior is a massive window for an adolescent undergoing rapid somatic growth and unpredictable growth spurts.

Action Required: The authors must discuss how developmental growth fluctuations over a 60-day window confound the "intentional" WL% reported by these young athletes.

Minor Comments

Table 2 Discrepancy: The row label says habitual WL/WR before competition (%) but the abstract and results text reference lower habitual weight regain relative to WL (WR/WL). Please standardize whether you are calculating WR divided by WL, or WL divided by WR, as the table label currently contradicts the text.

Table 3 and 4 Power: Given that the medalist group (n=46) and non-medalist group (n=79) have an uneven distribution, and many cells in the Chi-square/Phi analysis have frequencies < 5 (e.g., Diuretics, Laxatives, Diet pills), a standard Chi-square test is violated. Fisher’s Exact Test should be utilized and stated for these low-frequency categorical variables.

Reviewer #2: Dear Authors,

Thank you for the opportunity to review this interesting manuscript. The study addresses a relevant and timely topic in combat sports, providing valuable insights into weight loss practices and perceptions among national-level adolescent Sanda athletes. The manuscript is well organized, the methodology is clearly described, but the statistical analyses should be reported in greater detail and better justified according to the study design. The results provide meaningful information regarding the temporal structure of weight loss, weight regain control, and potential differences in strategic approaches between athletes with different competitive outcomes. The discussion is generally balanced and appropriately contextualized within the existing literature on rapid weight loss in combat sports. Overall, this manuscript contributes valuable evidence to the understanding of weight management practices among adolescent athletes competing in weight-class sports.

I believe the manuscript is suitable for publication after a few minor revisions:

• 154-156. The justification for not performing an a priori sample size calculation is acceptable for an exploratory study involving a limited population. However, the manuscript does not clearly describe the sampling strategy. Please specify whether all eligible athletes were invited to participate or whether a convenience sample was used. The current wording ("all available athletes accessible") is ambiguous and should not be interpreted as implying random or representative sampling.

Being a hard-to-reach population does not justify the absence of an a priori sample size calculation.

• 172. What is the scientific validity of the RWLQ developed by Zhong et al.? Specifically, what are its psychometric properties (e.g., Cronbach's alpha), and what response format or rating scale does it use?

• 185. For which variable was the t-test applied, given that almost all variables did not meet the assumption of normality?

• Table 1- Table 2. In the tables, the mean and standard deviation should not be reported for variables showing a highly skewed distribution, as the standard deviation values exceed the mean, indicating that these statistics may provide a misleading description of the data. Therefore, the median and interquartile range (IQR) should be reported, as they are considered more appropriate indicators of central tendency and dispersion.

**********

what does this mean?). If published, this will include your full peer review and any attached files.

If you choose “no”, your identity will remain anonymous but your review may still be made public.

**Do you want your identity to be public for this peer review?**  For information about this choice, including consent withdrawal, please see our Privacy Policy

Reviewer #1: No

Reviewer #2: No

**********

[NOTE: If reviewer comments were submitted as an attachment file, they will be attached to this email and accessible via the submission site. Please log into your account, locate the manuscript record, and check for the action link "View Attachments". If this link does not appear, there are no attachment files.]

To ensure your figures meet our technical requirements, please review our figure guidelines: https://journals.plos.org/plosone/s/figures

You may also use PLOS’s free figure tool, NAAS, to help you prepare publication quality figures: https://journals.plos.org/plosone/s/figures#loc-tools-for-figure-preparation.

NAAS will assess whether your figures meet our technical requirements by comparing each figure against our figure specifications.

# Author comment: Author response to Decision Letter 1

**DOI:** 10.1371/journal.pone.0359088.r002

30 Jul 2026

(Manuscript ID: PONE-D-26-30122)

Dear Editor and Reviewers:

Thank you for your positive response and for providing the reviewers’ comments concerning our manuscript entitled “Differences in weight loss strategies between medalists and non-medalists in national-level adolescent Sanda athletes”.

The reviewer’s comments have significantly improved our paper. We highlighted the changes of the manuscript within the document by tracked change.

Response to Reviewer 1:

Reviewer:

1. Statistical Significance vs. Practical Meaningfulness (Missing Effect Sizes)

The Issue: In Table 2, you report a statistically significant difference in habitual WL% between medalists (9.6 ± 3.5%) and non-medalists (8.1 ± 4.0%) with a p-value of 0.043. The mean difference is a mere 1.5%, and the 95% confidence interval (0.05% to 2.86%) barely scratches the surface of zero. For a 60kg adolescent athlete, a 1.5% difference equates to roughly 900g.

The Critique: Relying solely on a fragile p-value (p = 0.043) without reporting effect sizes (e.g., Cohen’s d or Hedges’ g) is insufficient for a journal of PLOS ONE’s caliber. A 900g absolute difference in habitual weight cutting across months cannot be logically or biochemically crowned as a "distinct strategic pattern" or a "decisive model for competitive success".

Action Required: Report the exact effect sizes for all significant continuous variables (habitual WL%, early phase WL%, and WR/WL). The discussion must be toned down to reflect that the actual magnitude of difference between the two groups is marginal at best.

Reply: We appreciate the reviewer's thoughtful comment regarding the distinction between statistical significance and practical significance. Following this suggestion, we have revised the manuscript in several ways.

First, we now report effect sizes for all statistically significant continuous variables. Specifically, Cohen's d was reported for the normally distributed variable (habitual WL%), whereas rank-biserial correlation (r_rb_) was reported for variables analyzed using the Mann–Whitney U test. These effect sizes indicated that the observed between-group differences were small. We agree that statistical significance should be interpreted together with effect size and clinical/practical relevance rather than P values alone.

Second, we have revised the Discussion to explicitly acknowledge the modest magnitude of the observed differences. Statements describing medalists as exhibiting a "distinct strategic pattern" or implying a "decisive model for competitive success" have been removed or substantially toned down. The revised manuscript now emphasizes that only three modest differences were observed, that the corresponding effect sizes were small, and that these findings should not be interpreted as evidence of a superior or causal WL strategy.

Finally, the Abstract and Conclusion have also been revised to ensure that the interpretation of the findings is appropriately cautious and reflects the limited magnitude of the observed differences.

Reviewer:

2. Flawed Causal Inference from Cross-Sectional Design

The Issue: The conclusion states that "competitive differences may be associated not with the specific WL methods used, but with how WL is structured and regulated over time," framing a structured WL strategy as a driver/cause of winning a medal.

The Critique: This is a classic cross-sectional study using purposive sampling. You cannot infer directionality. It is highly plausible that medalists are simply more experienced, have competed longer, or belong to better-funded elite training bases where coaches enforce stricter monitoring, which consequently leads to better weight management. The "strategy" could be a byproduct of being an elite medalist, not the cause of it.

Action Required: Reword the Abstract, Discussion, and Conclusion to completely eliminate any causal or predictive undertones. Explicitly acknowledge the limitation of reverse causality in the limitations section.

Reply: We appreciate the reviewer's important comment regarding causal inference. We agree that the cross-sectional design of the present study does not permit conclusions regarding the direction of the observed associations.

Accordingly, we have revised the Abstract, Discussion, and Conclusion to remove causal or predictive language throughout the manuscript. Expressions implying that habitual WL strategies may "drive" or explain competitive success have been replaced with more neutral descriptions emphasizing observed between-group differences only.

In addition, we have expanded the limitations section to explicitly acknowledge the possibility of reverse causality. Specifically, we now state that the observed differences may reflect greater competitive experience or other unmeasured factors associated with successful athletes, rather than habitual WL practices contributing to competitive success.

Reviewer:

3. The "Exploratory Nature" Excuse and Type I Error Inflation

The Issue: In the Statistical Analyses section, the authors state that "given the exploratory nature of the study and the absence of a single primary outcome, no formal adjustment for multiple comparisons was applied."

The Critique: You ran dozens of independent t-tests, Mann-Whitney U tests, and Chi-square tests across Tables 1, 2, 3, and 4. When conducting dozens of simultaneous hypotheses tests at alpha = 0.05, the family-wise error rate (the probability of falsely detecting at least one significant difference due to sheer randomness) inflates exponentially. Your borderline p-values (p = 0.043 for habitual WL%) are highly susceptible to being false positives (Type I error).

Action Required: Apply a formal correction for multiple comparisons (e.g., Benjamini-Hochberg False Discovery Rate or Bonferroni) to verify if these differences survive. If they do not, the core narrative of the paper must change.

Reply: We appreciate the reviewer's concern regarding inflation of Type I error. We acknowledge that multiple statistical comparisons increase the probability of false-positive findings.

However, the present study was designed as an exploratory cross-sectional investigation without a single predefined primary outcome. Rather than testing multiple hypotheses for one endpoint, the analyses examined different domains of habitual WL practices (Age began WL, Highest WL (%), Habitual WL (%), Habitual WL during 9-60, 2-8, 1 days prior to weigh-in (%), habitual WR before competition (%), habitual WL/WR, habitual WR after competition (%), Number of WL in the last year), specific methods, sources of influence, and perceptions. Therefore, we did not apply a formal family-wise error correction, as such adjustments may be overly conservative in exploratory observational research and substantially increase the risk of Type II error.

Instead, we have strengthened the manuscript by (i) reporting effect sizes for all statistically significant continuous variables, (ii) substantially moderating the interpretation of the findings throughout the Abstract, Discussion, and Conclusion, and (iii) explicitly acknowledging in the Limitations that no formal adjustment for multiple comparisons was performed and that the statistically significant findings should be regarded as exploratory and confirmed in future studies.

Reviewer:

4. Methodological Disconnect in the "Multi-Day Competition" Logic

The Issue: In the Discussion, the authors argue that lower habitual WR/WL in medalists reflects a controlled approach tailored to the demands of "multi-day Sanda competitions" where body mass stability across consecutive days is crucial.

The Critique: This is a massive leap in logic. Your questionnaire captured habitual weight regain relative to weight loss before the competition cycle (pre-weigh-in phase). It does not capture the actual day-to-day weight fluctuations or acute post-weigh-in fluid/energy titration during a multi-day tournament. You are using pre-competition recall data to justify an in-competition physiological strategy.

Action Required: Either remove the speculative multi-day competition hypothesis or clearly state that the study lacked the granulatory data (such as daily weigh-in charts during tournaments) to substantiate this claim.

Reply: Thank you for this insightful comment. We agree that our original discussion overstated the implications of the observed WR/WL difference for body mass regulation during multi-day competitions. We acknowledge that the study did not collect repeated day-to-day body mass measurements during competitions. Therefore, the present data cannot directly support inferences regarding body mass fluctuations or recovery dynamics across consecutive competition days. Accordingly, we have revised this section of the Discussion to focus on the observed difference in habitual WR relative to WL, removed the stronger interpretation regarding multi-day competition physiology, and explicitly state that any explanation for this finding remains speculative and requires confirmation in future longitudinal studies..

Reviewer:

5. Recall Bias and Developmental Vulnerability of Adolescents

The Issue: The study heavily relies on retrospective self-reporting via the RWLQ in a very young cohort (12-17 years old).

The Critique: Asking a 14-year-old athlete to recall their exact "habitual weight loss percentage during the 9-60 days prior to weigh-in" introduces massive recall bias. Sixty days prior is a massive window for an adolescent undergoing rapid somatic growth and unpredictable growth spurts.

Action Required: The authors must discuss how developmental growth fluctuations over a 60-day window confound the "intentional" WL% reported by these young athletes.

Reply: Thank you for this valuable comment. We agree that retrospective self-report may introduce recall bias, particularly in adolescent athletes. However, we would like to clarify that participants were not asked to recall exact weight loss percentages. Instead, the questionnaire asked athletes to report the amount of body mass (kg) they typically lost and regained before competition, which is a familiar aspect of their routine weight management. Weight loss percentages were subsequently calculated by the researchers based on the reported values. Nevertheless, we acknowledge that natural growth and developmental changes during adolescence, together with the retrospective nature of the questionnaire, may have influenced the estimation of habitual WL over the 60-day pre-competition period. This limitation has now been added to the Discussion.

Reviewer:

6. Table 2 Discrepancy: The row label says habitual WL/WR before competition (%) but the abstract and results text reference lower habitual weight regain relative to WL (WR/WL). Please standardize whether you are calculating WR divided by WL, or WL divided by WR, as the table label currently contradicts the text.

Reply: Thank you for your comment. We revised it.

Reviewer:

7. Table 3 and 4 Power: Given that the medalist group (n=46) and non-medalist group (n=79) have an uneven distribution, and many cells in the Chi-square/Phi analysis have frequencies < 5 (e.g., Diuretics, Laxatives, Diet pills), a standard Chi-square test is violated. Fisher’s Exact Test should be utilized and stated for these low-frequency categorical variables.

Reply: Thank you for this helpful comment. We agree that Pearson's chi-square test may not be appropriate for contingency tables with sparse expected cell frequencies. Accordingly, categorical variables that violated the assumptions of Pearson's chi-square test were reanalyzed using Fisher–Freeman–Halton exact tests. The Methods section has been revised accordingly. The exact tests produced results consistent with the original analyses.

Again, we would like to thank you again for reviewers’ comments. Your valuable opinions have improved the quality of the manuscript. We hope we have satisfactorily addressed the comments that were made.

Response to Reviewer 2:

Reviewer:

154-156. The justification for not performing an a priori sample size calculation is acceptable for an exploratory study involving a limited population. However, the manuscript does not clearly describe the sampling strategy. Please specify whether all eligible athletes were invited to participate or whether a convenience sample was used. The current wording ("all available athletes accessible") is ambiguous and should not be interpreted as implying random or representative sampling. Being a hard-to-reach population does not justify the absence of an a priori sample size calculation.

Reply: Thank you for this valuable comment. We agree that the original description of the sampling strategy was insufficiently clear. The Methods section has been revised to explicitly state that purposive and convenience sampling were used, and that all athletes meeting the inclusion criteria who were accessible through the participating sports teams during the recruitment period were invited to participate. We also revised the statement regarding the absence of an a priori sample size calculation to avoid implying that the hard-to-reach nature of the population justified this decision. Instead, we clarify that recruitment continued throughout the predefined data collection period to maximize participation in this exploratory cross-sectional study.

Reviewer:

172. What is the scientific validity of the RWLQ developed by Zhong et al.? Specifically, what are its psychometric properties (e.g., Cronbach's alpha), and what response format or rating scale does it use?

Reply: Thank you for this helpful comment. We apologize for the ambiguity in the original manuscript. The questionnaire used in this study was not newly developed by Zhong et al., but was an adapted version of the Rapid Weight Loss Questionnaire (RWLQ) originally developed and validated by Artioli et al (ICC > 0.90 for all questions whose answers were parametric, Cronbach’s a was 0.98 for scores obtained between test and retest). Zhong et al. subsequently adapted the questionnaire for Chinese Sanda athletes by adding several descriptive items while retaining the original questionnaire structure. We have revised the Methods section to clarify the origin of the questionnaire and the nature of the adaptation.

The original RWLQ has established psychometric validity, whereas the adaptations introduced by Zhong et al. consisted of additional descriptive questions rather than modifications to the validated measurement structure. We have also clarified the questionnaire structure and response formats in the Methods section to improve transparency.

Reference: Artioli, G.G., Scagliusi, F., Kashiwagura, D., Franchini, E., Gualano, B. and Junior, A.L. (2010), Development, validity and reliability of a questionnaire designed to evaluate rapid weight loss patterns in judo players. Scandinavian Journal of Medicine & Science in Sports, 20: e177-e187. https://doi.org/10.1111/j.1600-0838.2009.00940.x

Reviewer:

185. For which variable was the t-test applied, given that almost all variables did not meet the assumption of normality?

Reply: Thank you for this helpful comment. We agree that the original description of the statistical analyses did not clearly indicate which variable satisfied the assumption of normality. We have revised the Statistical Analysis section to explicitly state that only habitual WL (%) was normally distributed and was therefore analyzed using an independent-samples t-test, whereas all other continuous variables were analyzed using the Mann–Whitney U test.

Reviewer:

Table 1- Table 2. In the tables, the mean and standard deviation should not be reported for variables showing a highly skewed distribution, as the standard deviation values exceed the mean, indicating that these statistics may provide a misleading description of the data. Therefore, the median and interquartile range (IQR) should be reported, as they are considered more appropriate indicators of central tendency and dispersion.

Reply: Thank you for this valuable suggestion. We agree that mean ± SD is not appropriate for variables with skewed distributions. Accordingly, we have revised Tables 1 and 2 so that normally distributed continuous variables are presented as mean ± SD, whereas non-normally distributed continuous variables are presented as median (IQR). This presentation is now consistent with the distribution of each variable and the statistical methods described in the revised Methods section.

Again, we would like to thank you again for comments, which improves the quality of the manuscript.

Attachment Submitted filename: Response to Reviewers.docx

# Peer review: Decision Letter 1

**DOI:** 10.1371/journal.pone.0359088.r003

**Authors:** Stefano Amatori

17 Aug 2026

Dear Dr. Zhong,

Thank you for submitting your manuscript to PLOS ONE. After careful consideration, we feel that it has merit but does not fully meet PLOS ONE’s publication criteria as it currently stands. Therefore, we invite you to submit a revised version of the manuscript that addresses the points raised during the review process.

Particularly, the Reviewer #1 has raised some important methodological concerns which must be addressed before the manuscript can be considered further.

Please submit your revised manuscript by Oct 16 2026 11:59PM. If you will need more time than this to complete your revisions, please reply to this message or contact the journal office at plosone@plos.org. When you're ready to submit your revision, log on to https://www.editorialmanager.com/pone/ and select the 'Submissions Needing Revision' folder to locate your manuscript file.

- A letter that responds to each point raised by the academic editor and reviewer(s). You should upload this letter as a separate file labeled 'Response to Reviewers'.
- A marked-up copy of your manuscript that highlights changes made to the original version. You should upload this as a separate file labeled 'Revised Manuscript with Track Changes'.
- An unmarked version of your revised paper without tracked changes. You should upload this as a separate file labeled 'Manuscript'.

If you would like to make changes to your financial disclosure, please include your updated statement in your cover letter. Guidelines for resubmitting your figure files are available below the reviewer comments at the end of this letter.

If applicable, we recommend that you deposit your laboratory protocols in protocols.io to enhance the reproducibility of your results. Protocols.io assigns your protocol its own identifier (DOI) so that it can be cited independently in the future. For instructions see: https://journals.plos.org/plosone/s/submission-guidelines#loc-laboratory-protocols. Additionally, PLOS ONE offers an option for publishing peer-reviewed Lab Protocol articles, which describe protocols hosted on protocols.io. Read more information on sharing protocols at https://plos.org/protocols?utm_medium=editorial-email&utm_source=authorletters&utm_campaign=protocols.

As the corresponding author, your ORCID iD is verified in the submission system and will appear in the published article. PLOS supports the use of ORCID, and we encourage all coauthors to register for an ORCID iD and use it as well. Please encourage your coauthors to verify their ORCID iD within the submission system before final acceptance, as unverified ORCID iDs will not appear in the published article. *Only*  the individual author can complete the verification step; PLOS staff *cannot*  verify ORCID iDs on behalf of authors.

We look forward to receiving your revised manuscript.

Kind regards,

Stefano Amatori, Ph.D.

Academic Editor

PLOS One

Journal Requirements:

If the reviewer comments include a recommendation to cite specific previously published works, please review and evaluate these publications to determine whether they are relevant and should be cited. There is no requirement to cite these works unless the editor has indicated otherwise.

[Note: HTML markup is below. Please do not edit.]

Reviewers' comments:

Reviewer's Responses to Questions

**Comments to the Author**

Reviewer #1: All comments have been addressed

Reviewer #2: All comments have been addressed

**********

2. Is the manuscript technically sound, and do the data support the conclusions?

Reviewer #1: No

Reviewer #2: Partly

**********

3. Has the statistical analysis been performed appropriately and rigorously?

Reviewer #1: No

Reviewer #2: Yes

**********

4. Have the authors made all data underlying the findings in their manuscript fully available?

The PLOS Data policy

Reviewer #1: Yes

Reviewer #2: Yes

**********

5. Is the manuscript presented in an intelligible fashion and written in standard English?

Reviewer #1: No

Reviewer #2: Yes

**********

Reviewer #1: ## Review Comments to the Author

Thank you for the opportunity to review the revised manuscript. The authors have made several efforts to address the previous reviewer comments, and the manuscript is clearer in some respects. However, after reviewing the revised version, I do not consider the major methodological and interpretational concerns to have been adequately resolved. In my view, the manuscript still does not provide sufficiently rigorous evidence to support its principal conclusions regarding differences in weight-loss strategies between medalists and non-medalists. I therefore cannot recommend publication in its current form.

### Major Concern 1. The statistical analysis remains insufficiently rigorous

The manuscript examines a large number of weight-loss-related outcomes, including the timing and magnitude of weight loss, weight regain, ratios, frequency, methods, sources of influence, and perceptions. Nevertheless, the analysis appears to rely predominantly on multiple unadjusted group comparisons, with no appropriate control for the substantial multiplicity of statistical testing.

The authors justify the absence of multiplicity adjustment by describing the study as exploratory. However, an exploratory designation does not eliminate the risk of inflated type I error. This is particularly important because the statistically significant findings appear to be relatively few and are associated with modest effect sizes and p-values close to the conventional significance threshold. Consequently, it is difficult to determine whether the reported significant findings represent robust associations or false-positive findings arising from the large number of comparisons.

At minimum, the authors should clearly distinguish prespecified primary outcomes from exploratory analyses and provide an appropriate sensitivity analysis for multiple testing. Without such an analysis, the statistical evidence for the manuscript's principal claims remains weak.

### Major Concern 2. Potential confounding has not been adequately addressed

The central comparison is between medalists and non-medalists, yet the manuscript does not adequately account for variables that could plausibly influence both competitive achievement and weight-loss practices. Relevant factors may include age, sex, competitive experience, number of competitions, training environment, and other characteristics related to competitive exposure.

The decision not to adjust for sex because the medalist and non-medalist groups did not differ statistically in sex distribution is not a sufficient methodological justification. The absence of a statistically significant baseline group difference does not establish that a variable cannot confound an association.

More importantly, the manuscript reports that competition-related characteristics are associated with weight-loss practices, yet these factors are not incorporated into an adjusted analysis of medalist status. Therefore, the observed differences cannot confidently be attributed to medalist status itself.

The authors should either conduct appropriately adjusted analyses or substantially reduce the strength of the conclusions and explicitly frame the findings as unadjusted exploratory associations.

### Major Concern 3. The temporal relationship between medal achievement and reported weight-loss behavior is unclear

The manuscript defines medalists based on whether an athlete had achieved a national-level medal during their career, whereas the weight-loss variables represent retrospectively reported habitual practices. This creates an important temporal ambiguity.

It is unclear whether the reported weight-loss practices occurred before or after the athlete's medal achievement. Therefore, the data cannot establish that a particular weight-loss strategy contributed to competitive success. Conversely, the possibility that competitive experience or achievement influenced subsequent weight-loss behavior cannot be excluded.

Accordingly, statements suggesting that medalists "adopt" particular strategies or that specific weight-loss patterns may contribute to competitive success are not adequately supported by the study design.

The conclusions should be restricted to associations between reported weight-loss characteristics and a history of medal achievement.

### Major Concern 4. Several interpretations extend beyond what was actually measured

The manuscript interprets the greater proportion of weight loss occurring during the earlier 9–60-day period among medalists as evidence of a "more gradual" or better-organized weight-loss strategy. However, the questionnaire does not directly establish that these athletes followed a controlled or physiologically safer gradual weight-loss protocol.

Similarly, the lower WR/WL ratio among medalists should not be interpreted as evidence of "tighter control" of post-weigh-in recovery. The reported difference in the ratio appears to be driven substantially by the denominator, because absolute weight regain itself was not significantly different between groups.

The discussion should therefore distinguish carefully between what was directly measured and what is inferred from those measurements.

### Major Concern 5. Claims regarding multi-day competition physiology are not sufficiently supported by the collected data

The manuscript discusses multi-day Sanda competition and suggests that limiting post-weigh-in weight regain may contribute to smaller body-mass fluctuations and more stable physiological conditions. However, the present study did not directly measure day-to-day body-mass fluctuations, hydration status, physiological stability, performance, or recovery during competition.

These statements therefore go beyond the empirical data presented in this study. They may be reasonable hypotheses for future research, but they should not be presented as interpretations supported by the current dataset.

### Major Concern 6. The practical implications are stronger than warranted by the observational design

The manuscript suggests that earlier and more gradual weight loss may be beneficial and implies that the temporal organization of weight loss may be an important practical target.

However, this is a retrospective observational study without a direct performance outcome or prospective assessment of health consequences. The finding that medalists reported a different temporal distribution of weight loss does not demonstrate that earlier or more gradual weight loss improves performance, health, or competitive success.

The practical implications should therefore be substantially moderated. The data support the identification of an association or behavioral pattern, not a recommendation that one weight-loss strategy is superior.

### Major Concern 7. The manuscript's central terminology does not fully correspond to the evidence

The title and several parts of the manuscript refer to "weight-loss strategies." However, the study does not demonstrate that medalists and non-medalists differed substantially in specific weight-loss methods. Rather, the principal differences appear to concern selected characteristics such as the timing or magnitude of reported weight loss and weight regain.

The terminology should therefore be reconsidered so that the title and conclusions accurately reflect the observational and self-reported nature of the data.

### Major Concern 8. The revised manuscript still contains substantial editing and language problems

The revised manuscript contains numerous unresolved track-change remnants, duplicated or contradictory wording, typographical errors, and grammatical problems. Examples include constructions such as "standardized similar among between," "includingNevertheless," "Hhabitual WLWR/WLR," and other incomplete or duplicated phrases.

These issues substantially reduce readability and create ambiguity in several methodological and interpretational statements. Given that PLOS ONE does not provide substantive copyediting after acceptance, the manuscript requires a careful and complete language and formatting revision before it can be considered publication-ready.

### Overall assessment

The study addresses a potentially relevant topic in adolescent combat-sport athletes, and the dataset may provide useful descriptive information regarding weight-loss practices. However, the principal conclusions currently rely on multiple unadjusted statistical comparisons, do not adequately address potential confounding or temporal ambiguity, and extend the interpretation beyond what was directly measured.

Importantly, these are not merely editorial issues that can be resolved through minor textual revision. The major concerns involve the analytical framework and the interpretation of the primary findings. Unless the authors substantially reanalyze the data and appropriately revise the claims, the current evidence is insufficient to support the manuscript's central conclusions.

Reviewer #2: (No Response)

**********

what does this mean?). If published, this will include your full peer review and any attached files.

If you choose “no”, your identity will remain anonymous but your review may still be made public.

**Do you want your identity to be public for this peer review?**  For information about this choice, including consent withdrawal, please see our Privacy Policy

Reviewer #1: No

Reviewer #2: No

**********

[NOTE: If reviewer comments were submitted as an attachment file, they will be attached to this email and accessible via the submission site. Please log into your account, locate the manuscript record, and check for the action link "View Attachments". If this link does not appear, there are no attachment files.]

To ensure your figures meet our technical requirements, please review our figure guidelines: https://journals.plos.org/plosone/s/figures

You may also use PLOS’s free figure tool, NAAS, to help you prepare publication quality figures: https://journals.plos.org/plosone/s/figures#loc-tools-for-figure-preparation.

NAAS will assess whether your figures meet our technical requirements by comparing each figure against our figure specifications.

# Author comment: Author response to Decision Letter 2

**DOI:** 10.1371/journal.pone.0359088.r004

8 Sep 2026

(Manuscript ID: PONE-D-26-30122)

Dear Editor and Reviewers:

Thank you for providing the reviewers’ comments concerning our manuscript entitled “Differences in weight loss strategies between medalists and non-medalists in national-level adolescent Sanda athletes”.

The reviewer’s comments have significantly improved our paper. We highlighted the changes of the manuscript within the document by tracked change (yellow background).

Response to Reviewer 1:

Reviewer:

Major Concern 1. The statistical analysis remains insufficiently rigorous

The manuscript examines a large number of weight-loss-related outcomes, including the timing and magnitude of weight loss, weight regain, ratios, frequency, methods, sources of influence, and perceptions. Nevertheless, the analysis appears to rely predominantly on multiple unadjusted group comparisons, with no appropriate control for the substantial multiplicity of statistical testing.

The authors justify the absence of multiplicity adjustment by describing the study as exploratory. However, an exploratory designation does not eliminate the risk of inflated type I error. This is particularly important because the statistically significant findings appear to be relatively few and are associated with modest effect sizes and p-values close to the conventional significance threshold. Consequently, it is difficult to determine whether the reported significant findings represent robust associations or false-positive findings arising from the large number of comparisons.

At minimum, the authors should clearly distinguish prespecified primary outcomes from exploratory analyses and provide an appropriate sensitivity analysis for multiple testing. Without such an analysis, the statistical evidence for the manuscript's principal claims remains weak.

Reply: Thank you for this important comment. We agree that the large number of statistical comparisons increases the risk of inflated type I error. We therefore conducted a Benjamini–Hochberg FDR sensitivity analysis, with related outcomes grouped into four conceptual outcome families. The study was exploratory in nature and did not prespecify a single primary WL outcome.

None of the three nominally significant associations identified in the original unadjusted analyses (habitual WL%, habitual WL% during the 9–60 days prior to weigh-in, and habitual WR/WL before competition) remained statistically significant after FDR adjustment. We have therefore revised the Abstract, Results, Discussion, and Conclusion to clearly distinguish these unadjusted findings as exploratory and hypothesis-generating, rather than robust between-group differences. The revised manuscript now emphasizes that the available data do not provide evidence of systematic differences in WL practices or perceptions according to medal achievement.

Reviewer:

Major Concern 2. Potential confounding has not been adequately addressed

The central comparison is between medalists and non-medalists, yet the manuscript does not adequately account for variables that could plausibly influence both competitive achievement and weight-loss practices. Relevant factors may include age, sex, competitive experience, number of competitions, training environment, and other characteristics related to competitive exposure.

The decision not to adjust for sex because the medalist and non-medalist groups did not differ statistically in sex distribution is not a sufficient methodological justification. The absence of a statistically significant baseline group difference does not establish that a variable cannot confound an association.

More importantly, the manuscript reports that competition-related characteristics are associated with weight-loss practices, yet these factors are not incorporated into an adjusted analysis of medalist status. Therefore, the observed differences cannot confidently be attributed to medalist status itself.

The authors should either conduct appropriately adjusted analyses or substantially reduce the strength of the conclusions and explicitly frame the findings as unadjusted exploratory associations.

Reply: Thank you for this important comment. We agree that potential confounding should be considered when comparing medalists and non-medalists. In response, we substantially reduced the strength of the interpretation rather than attributing the observed unadjusted associations to medalist status.

Importantly, after the Benjamini–Hochberg FDR sensitivity analysis, none of the originally observed WL-related associations remained statistically significant. Accordingly, the revised manuscript no longer presents medalist status as being associated with specific WL characteristics, and all unadjusted findings are explicitly described as nominal, exploratory, and hypothesis-generating. We therefore did not perform a post hoc multivariable adjustment analysis, as there was no longer a statistically significant association that we sought to model as an independent effect of medalist status. Nevertheless, we acknowledge that residual confounding by sex, age, competitive experience, number of competitions, and other unmeasured factors cannot be excluded and have added this limitation to the revised manuscript.

Reviewer:

Major Concern 3. The temporal relationship between medal achievement and reported weight-loss behavior is unclear

The manuscript defines medalists based on whether an athlete had achieved a national-level medal during their career, whereas the weight-loss variables represent retrospectively reported habitual practices. This creates an important temporal ambiguity.

It is unclear whether the reported weight-loss practices occurred before or after the athlete's medal achievement. Therefore, the data cannot establish that a particular weight-loss strategy contributed to competitive success. Conversely, the possibility that competitive experience or achievement influenced subsequent weight-loss behavior cannot be excluded.

Accordingly, statements suggesting that medalists "adopt" particular strategies or that specific weight-loss patterns may contribute to competitive success are not adequately supported by the study design.

The conclusions should be restricted to associations between reported weight-loss characteristics and a history of medal achievement.

Reply: Thank you for this important comment. We agree that the cross-sectional design and retrospective assessment of WL practices do not allow us to establish the temporal sequence between WL behavior and medal achievement. We have substantially revised the interpretation and conclusions throughout the manuscript and removed statements suggesting that medalists adopted particular WL strategies or that specific WL patterns contributed to competitive success. We also acknowledged this limitation in the limitation section.

Reviewer:

Major Concern 4. Several interpretations extend beyond what was actually measured

The manuscript interprets the greater proportion of weight loss occurring during the earlier 9–60-day period among medalists as evidence of a "more gradual" or better-organized weight-loss strategy. However, the questionnaire does not directly establish that these athletes followed a controlled or physiologically safer gradual weight-loss protocol.

Similarly, the lower WR/WL ratio among medalists should not be interpreted as evidence of "tighter control" of post-weigh-in recovery. The reported difference in the ratio appears to be driven substantially by the denominator, because absolute weight regain itself was not significantly different between groups.

The discussion should therefore distinguish carefully between what was directly measured and what is inferred from those measurements.

Reply: Thank you for this important comment. We agree that these interpretations extended beyond what was directly measured by the questionnaire. We have therefore removed the descriptions of a “more gradual” or “better-organized” WL strategy and the interpretation of lower WR/WL as “tighter control” of post-weigh-in recovery. We now distinguish more clearly between the measured WL and WR variables and their possible interpretations. In addition, because these associations did not remain statistically significant after FDR adjustment, they are now presented only as nominal, exploratory findings rather than evidence of distinct WL or WR strategies.

Reviewer:

Major Concern 5. Claims regarding multi-day competition physiology are not sufficiently supported by the collected data

The manuscript discusses multi-day Sanda competition and suggests that limiting post-weigh-in weight regain may contribute to smaller body-mass fluctuations and more stable physiological conditions. However, the present study did not directly measure day-to-day body-mass fluctuations, hydration status, physiological stability, performance, or recovery during competition.

These statements therefore go beyond the empirical data presented in this study. They may be reasonable hypotheses for future research, but they should not be presented as interpretations supported by the current dataset.

Reply: Thank you for your comment. Regarding this part, we have already removed it in the previous round.

Reviewer:

Major Concern 6. The practical implications are stronger than warranted by the observational design

The manuscript suggests that earlier and more gradual weight loss may be beneficial and implies that the temporal organization of weight loss may be an important practical target.

However, this is a retrospective observational study without a direct performance outcome or prospective assessment of health consequences. The finding that medalists reported a different temporal distribution of weight loss does not demonstrate that earlier or more gradual weight loss improves performance, health, or competitive success.

The practical implications should therefore be substantially moderated. The data support the identification of an association or behavioral pattern, not a recommendation that one weight-loss strategy is superior.

Reply: Thank you for this important comment. We agree that the original practical implications were too strong for the observational design. We have substantially revised the practical interpretation and removed statements suggesting that earlier or more gradual WL is beneficial or should be recommended as a preferred strategy. Given that the observed WL-related associations did not remain statistically significant after FDR adjustment, the revised manuscript does not propose any WL pattern as superior or performance-enhancing.

Reviewer:

Major Concern 7. The manuscript's central terminology does not fully correspond to the evidence

The title and several parts of the manuscript refer to "weight-loss strategies." However, the study does not demonstrate that medalists and non-medalists differed substantially in specific weight-loss methods. Rather, the principal differences appear to concern selected characteristics such as the timing or magnitude of reported weight loss and weight regain.

The terminology should therefore be reconsidered so that the title and conclusions accurately reflect the observational and self-reported nature of the data.

Reply:

Thank you for this comment. We agree that the term “weight-loss strategies” could overstate what was measured in this observational, self-reported study. We have therefore revised the terminology throughout the manuscript, including the title, Abstract, Discussion, and Conclusion.

Reviewer:

Major Concern 8. The revised manuscript still contains substantial editing and language problems

The revised manuscript contains numerous unresolved track-change remnants, duplicated or contradictory wording, typographical errors, and grammatical problems. Examples include constructions such as "standardized similar among between," "includingNevertheless," "Hhabitual WLWR/WLR," and other incomplete or duplicated phrases.

These issues substantially reduce readability and create ambiguity in several methodological and interpretational statements. Given that PLOS ONE does not provide substantive copyediting after acceptance, the manuscript requires a careful and complete language and formatting revision before it can be considered publication-ready.

Reply: Thank you for pointing this out, and we sincerely apologize for the confusion and inconvenience. PLOS ONE requires both a tracked-changes version and a clean version for submission. In the previous submission, the tracked-changes Word file was converted to PDF in a way that retained both the original and revised text, which resulted in duplicated and apparently contradictory wording. We acknowledge that this substantially affected the readability of the manuscript.

In the present revision, we have carefully reviewed the entire manuscript, removed all Track Changes and edited remnants, and corrected grammatical, typographical, and formatting errors. The revised manuscript is presented as a clean version, with changes highlighted in yellow instead of using Track Changes. We have also carefully checked the manuscript to ensure that no duplicated or contradictory text remains.

Again, we would like to thank you again for reviewers’ comments. Your valuable opinions have improved the quality of the manuscript. We hope we have satisfactorily addressed the comments that were made.

Attachment Submitted filename: Response_to_Reviewers_auresp_2.docx

# Editor report: Decision Letter 2

**DOI:** 10.1371/journal.pone.0359088.r005

**Authors:** Stefano Amatori

9 Sep 2026

Differences in weight loss practices and perceptions between medalists and non-medalists in national-level adolescent Sanda athletes

PONE-D-26-30122R2

Dear Dr. Zhong,

We’re pleased to inform you that your manuscript has been judged scientifically suitable for publication and will be formally accepted for publication once it meets all outstanding technical requirements.

Within one week, you’ll receive an e-mail detailing the required amendments. When these have been addressed, you’ll receive a formal acceptance letter and your manuscript will be scheduled for publication.

An invoice will be generated when your article is formally accepted. Please note, if your institution has a publishing partnership with PLOS and your article meets the relevant criteria, all or part of your publication costs will be covered. Please make sure your user information is up-to-date by logging into Editorial Manager at Editorial Manager® and clicking the ‘Update My Information' link at the top of the page. For questions related to billing, please contact billing support.

If your institution or institutions have a press office, please notify them about your upcoming paper to help maximize its impact. If they’ll be preparing press materials, please inform our press team as soon as possible -- no later than 48 hours after receiving the formal acceptance. Your manuscript will remain under strict press embargo until 2 pm Eastern Time on the date of publication. For more information, please contact onepress@plos.org.

Kind regards,

Stefano Amatori, Ph.D.

Academic Editor

PLOS One

Additional Editor Comments (optional):

Reviewers' comments:

# Editor report: Acceptance letter

**DOI:** 10.1371/journal.pone.0359088.r006

**Authors:** Stefano Amatori

PONE-D-26-30122R2

PLOS One

Dear Dr. Zhong,

I'm pleased to inform you that your manuscript has been deemed suitable for publication in PLOS One. Congratulations! Your manuscript is now being handed over to our production team.

At this stage, our production department will prepare your paper for publication. This includes ensuring the following:

* All references, tables, and figures are properly cited

* All relevant supporting information is included in the manuscript submission,

* There are no issues that prevent the paper from being properly typeset

You will receive further instructions from the production team, including instructions on how to review your proof when it is ready. Please keep in mind that we are working through a large volume of accepted articles, so please give us a few days to review your paper and let you know the next and final steps.

Lastly, if your institution or institutions have a press office, please let them know about your upcoming paper now to help maximize its impact. If they'll be preparing press materials, please inform our press team within the next 48 hours. Your manuscript will remain under strict press embargo until 2 pm Eastern Time on the date of publication. For more information, please contact onepress@plos.org.

You will receive an invoice from PLOS for your publication fee after your manuscript has reached the completed accept phase. If you receive an email requesting payment before acceptance or for any other service, this may be a phishing scheme. Learn how to identify phishing emails and protect your accounts at https://explore.plos.org/phishing.

If we can help with anything else, please email us at customercare@plos.org.

Thank you for submitting your work to PLOS One and supporting open access.

Kind regards,

PLOS One Editorial Office Staff

on behalf of

Prof. Stefano Amatori

Academic Editor

PLOS One
